# Transcriptome-based network analysis reveals renal cell type-specific dysregulation of hypoxia-associated transcripts

**DOI:** 10.1038/s41598-017-08492-y

**Published:** 2017-08-17

**Authors:** Natallia Shved, Gregor Warsow, Felix Eichinger, David Hoogewijs, Simone Brandt, Peter Wild, Matthias Kretzler, Clemens D. Cohen, Maja T. Lindenmeyer

**Affiliations:** 1 Institute of Physiology and Division of Nephrology, University of Zurich, Zurich, Switzerland; 2 Department of Anatomy and Cell Biology, Universitätsmedizin Greifswald, Greifswald, Germany; Division of Theoretical Bioinformatics (B080), German Cancer Research Center (DKFZ), Heidelberg, Germany; 3 Department of Medicine, University of Michigan, Ann Arbor, Michigan USA; 4 Department of Medicine/Physiology, University of Fribourg, Fribourg, Switzerland; Institute of Physiology, University of Zurich, Zurich, Switzerland; 5 Institute of Pathology and Molecular Pathology, University Hospital Zurich, Zurich, Switzerland; 6 Nephrological Center, Medical Clinic and Policlinic IV, University of Munich, Munich, Germany

## Abstract

Accumulating evidence suggests that dysregulation of hypoxia-regulated transcriptional mechanisms is involved in development of chronic kidney diseases (CKD). However, it remains unclear how hypoxia-induced transcription factors (HIFs) and subsequent biological processes contribute to CKD development and progression. In our study, genome-wide expression profiles of more than 200 renal biopsies from patients with different CKD stages revealed significant correlation of HIF-target genes with eGFR in glomeruli and tubulointerstitium. These correlations were positive and negative and in part compartment-specific. Microarrays of proximal tubular cells and podocytes with stable HIF1α and/or HIF2α suppression displayed cell type-specific HIF1/HIF2-dependencies as well as dysregulation of several pathways. WGCNA analysis identified gene sets that were highly coregulated within modules. Characterization of the modules revealed common as well as cell group- and condition-specific pathways, GO-Terms and transcription factors. Gene expression analysis of the hypoxia-interconnected pathways in patients with different CKD stages revealed an increased dysregulation with loss of renal function. In conclusion, our data clearly point to a compartment- and cell type-specific dysregulation of hypoxia-associated gene transcripts and might help to improve the understanding of hypoxia, HIF dysregulation, and transcriptional program response in CKD.

## Introduction

Chronic kidney disease (CKD) is a major and rapidly increasing worldwide public health problem that is associated with an excessively increased cardiovascular risk profile, significant mortality and socioeconomic burden^1, 2^. Most CKDs are initiated by glomerular alterations. The pathogenesis of the glomerular insult can be variable, including diabetes, hypertension and glomerulonephritis^2–4^. With ongoing progression irreversible pathological processes occur in the tubulointerstitium resulting in the development of end-stage renal disease (ESRD). The best morphologic indicator of disease progression and development of ESRD is currently the degree of interstitial fibrosis^5^.

One of the mechanisms which has been implicated in the development of tissue fibrosis is hypoxia caused by an imbalance of blood perfusion and oxygen demand^6^. The cellular response to hypoxia is largely mediated by the hypoxia-inducible factors (HIF), heterodimeric transcription factors consisting of a labile oxygen-regulated α-subunit, including HIF1α, HIF2α and HIF3α and a constitutively expressed β-subunit (HIFβ)^7^. The HIF isoforms and regulators of HIF (prolyl hydroxylases) show partly cell type-specific distributions in the kidney. While HIF1α is mainly found in tubular cells, HIF2α is expressed essentially in endothelial and interstitial cells, as well as in some glomerular cells^8, 9^. HIF expression is not apparent in the normal renal medulla despite physiologically low oxygen tension. Upregulation of HIF occurs in response to reduced oxygen content of the blood and, moreover, tubular cells differ in their hypoxia HIF response capacity. This capacity is most pronounced in collecting duct, less in proximal tubules and limited in thick limb^8^.

For more than a decade the “chronic hypoxia hypothesis” links hypoxia to tubular damage in CKD, with hypoxia acting as the transmitter of glomerular injury to the tubulointerstitium^10^. According to this idea, the glomerular damage leads to reduced postglomerular flow and tubulointerstitial hypoxia with subsequent tubular injury, inflammation, fibrosis and capillary rarefaction. Accumulating data from *in vitro* and animal studies support the presence of hypoxia and its potential pathogenic role in the chronic deterioration of renal function. The group of Nangaku could demonstrate that hypoxia induces a myofibroblastic phenotype in tubular epithelial cells and that prolonged exposure to hypoxia leads to mitochondrial dysfunction and subsequent apoptosis^11, 12^. Higgins and colleagues found that activation of epithelial HIF1α signaling is associated with the development of CKD and might contribute to the development of interstitial fibrosis via the induction of ECM-modifying and lysyl oxidase genes^13^.

In humans, evidence remains unclear as studies show divergent results. Immunohistochemistry data from kidney biopsies of patients with diabetic nephropathy, IgA-nephropathy or polycystic kidney disease display an increased expression of HIF1α, used as an indirect marker for hypoxia^13–15^, suggesting thereby the presence of hypoxia in these diseases. Additionally, data from patients with nephrosclerosis indicate that hypoxia-associated processes seem not only to be involved in tubulointerstitial fibrosis, but might also contribute to glomerular damage via upregulation of CXCR4^16^. On the other hand patients with advanced stages of CKD show despite anemia an impaired expression of erythropoetin as well as reduced expression of vascular endothelial growth factor A (VEGFA), both genes known to be induced by hypoxia^15, 17^. Furthermore, recent BOLD-MRI studies measuring renal oxygenation in CKD patients gave discrepant findings on whether renal oxygenation is reduced in CKD patients or not^18, 19^.

Since hypoxia has been associated with fibrosis, renal cells indeed might face hypoxia in CKD and respond with a transcriptional program which could lead to progression of renal disease. So, the aim of the study was to analyze 1) whether an eGFR-dependent induction of HIF-target genes can be detected in kidney biopsies of patients with CKD as support for the chronic hypoxia hypothesis, 2) which relevance HIFs have in the dysregulation of hypoxia-associated gene products in different renal cells and 3) which additional regulatory mechanisms might be involved and might contribute to disease progression.

## Results

### eGFR Correlation of HIF-target genes

To investigate whether an eGFR-dependent induction of HIF-target genes can be detected in patients with CKD, glomerular and tubulointerstitial expression of 83 literature-derived HIF-target genes^20, 21^ in patients with different glomerulopathies were correlated to eGFR using Spearman correlation. From a total of 83 HIF-target genes, 24 correlated with eGFR in the tubulointerstitium and 18 correlated with eGFR in glomerular samples (correlation criteria: ISpearman’s rhoI >0.3; FDR-corrected p-value < 0.05; Table 1). In order to estimate the significance of this finding, bootstrap correlation analyses were performed on glomerular and tubulointerstitial gene expression data by selection of 10,000 sets of 83 randomly chosen genes and subsequent determination of the number of genes that correlated significantly with eGFR (same correlation criteria as above). As a result, the observed enrichment for hypoxia-associated genes being correlated with eGFR is very unlikely to have occurred just by chance for glomerular samples, which does not hold for the tubulointerstitium (Supplementary Figure 1A and B). The findings were robust with respect to gene selection when we used bootstrap sample analyses for randomly chosen subsets of 60%, 70%, 80%, and 90% of the 83 HIF-target genes (Supplementary Figure 1C and D). While age did not correlate with gene expression indicating no influence of age on eGFR-dependent induction of HIF-target genes (data not shown), stratification by gender revealed sex-specific differences in correlation of eGFR and HIF target gene expression (Supplementary Table 1, Supplementary Figure 1E–H).

**Table 1:** Spearman Correlation Analysis for selected HIF-target genes with eGFR.

Entrez Gene ID	Gene Symbol	eGFR (Glom)	eGFR (Tub)
		ρ	ρ
9429	ABCG2		**0.579**
226	ALDOA		−*0.366*
79365	BHLHE41	−*0.335*	**−0.424**
664	BNIP3		**0.489**
10370	CITED2		*0.325*
1356	CP		−*0.317*
7852	CXCR4	−*0.334*	**−0.504**
54541	DDIT4	−*0.356*	
1906	EDN1		**−0.485**
2023	ENO1	−*0.356*	−*0.372*
26355	FAM162A		*0.304*
2235	FECH		*0.376*
2597	GAPDH	−*0.321*	
3091	HIF1A	−0.402	**−0.484**
7184	HSP90B1		−*0.378*
3484	IGFBP1	−*0.383*	
3486	IGFBP3	−*0.383*	
3689	ITGB2	**−** ***0.346***	**−0.511**
4015	LOX	*0.339*	
4017	LOXL2	−*0.390*	−*0.323*
4170	MCL1	−*0.314*	**−0.471**
4233	MET		**−0.445**
4601	MXI1		**0.508**
10397	NDRG1	*0.341*	**0.449**
4878	NPPA		*0.338*
5209	PFKFB3	**−0.441**	**−0.438**
5366	PMAIP1	−*0.316*	−*0.310*
6095	RORA		**0.418**
5054	SERPINE1	−*0.380*	
7037	TFRC	−*0.368*	
7422	VEGFA		**0.579**
7490	WT1	*0.338*	

Bold: ρ > І0.4І, adjusted p < 0.05; Italic: І0.4І > ρ > І0.3І, adjusted p < 0.05.

Interestingly, we did not see a continuous induction of the HIF-target genes which would result in a negative correlation with eGFR, but rather observed correlations that were both positive and negative and in part compartment-specific.

### Evaluation of selected HIF-targets by immunohistochemistry

**Figure 1:**
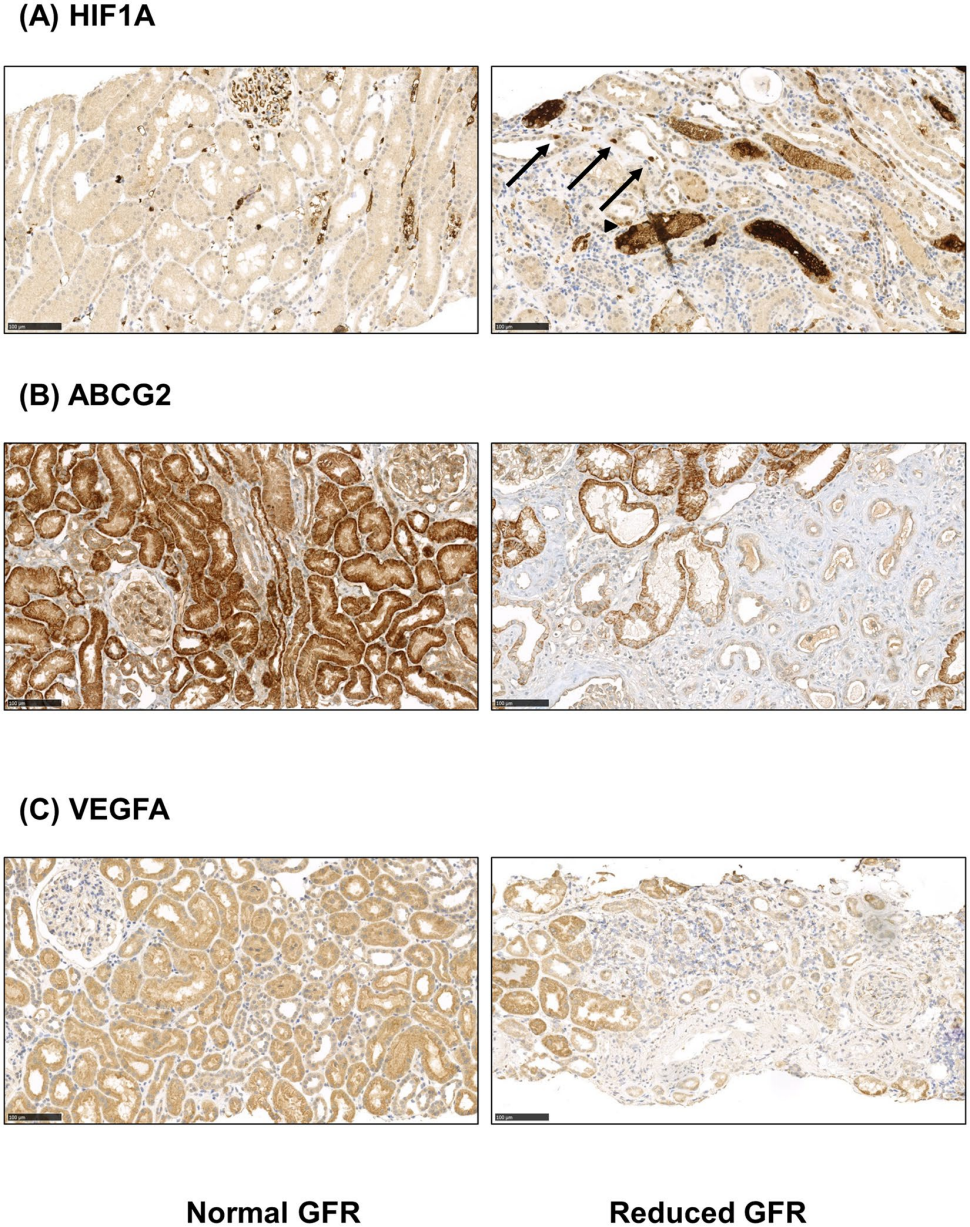
Immunohistochemistry of HIF1α, VEGFA and ABCG2. Sections from paraffin embedded kidney biopsies with chronic kidney disease (normal (left) and reduced eGFR (right)) were stained with antibodies against HIF1α (**A**), ATP binding cassette subfamily G member 2 (ABCG2) (**B**), and vascular endothelial growth factor A (VEGFA) (**C**). (**A**) No specific HIF1α staining could be detected in kidney sections with normal eGFR, score negative (0). Only some unspecific intravascular background staining is seen (left). In kidneys from patients with severely reduced eGFR, some nuclear (→) and cytoplasmic (►) tubular staining is detected, score weak (1) (right). (**B**) ABCG2 showing strong (score 2) expression in the tubulointerstitial compartment of kidneys with normal eGFR (left) in contrast to weak (score 1) staining in samples with reduced eGFR (right). (**C**) Similar stainig pattern for VEGFA, strong (score 2) expression in the tubulointerstitial compartment of kidneys with normal eGFR (left) in contrast to weak (score 1) staining in samples with reduced eGFR (right).

For evaluation of protein expression in human biopsies, immunohistochemistry of HIF1α, which showed a negative correlation with eGFR on transcriptomic level, and ATP binding cassette subfamily G member 2 (ABCG2) as well as VEGFA, both positively correlating with eGFR, was performed on a set of kidney biopsies of patients with CKD with normal and reduced eGFR (Fig. 1, Supplementary Figure 2). In patients with normal eGFR, no nuclear or cytoplasmic staining of HIF1α was detected in the glomerular or tubular compartment (score 0), only erythrocytes showed unspecific staining. In diseased kidneys with reduced renal function an increased nuclear and cytoplasmic expression (score 1) of HIF1α was found in tubular (p = 0.036) and to a lesser extent in glomerular cells (p = 0.007) which is in accordance with the transcriptomic correlation analysis and data from Higgins *et al*.^13^ (Fig. 1A, Supplementary Figure 2C and D). For ABCG2, a xenobiotic transporter, strong protein expression (score 2) could be detected in tubular epithelial cells in kidneys with normal eGFR, while the staining was clearly less intensive (weak, score 1) in some diseased kidneys with reduced eGFR (p < 0.001) (Fig. 1B, Supplementary Figure 2E). The angiogenic factor VEGFA showed strong tubular (score 2) and only very weak staining in the glomerulus of the kidney with normal eGFR, and was less intensive (weak, score 1) in the tubulointerstitium in a subset of diseased kidneys with reduced eGFR (p = 0.001) (Fig. 1C, Supplementary Figure 2F). Overall, immunoreactivity levels of selected HIF-target genes in diseased kidney tissue with normal and reduced eGFR showed patterns comparable to the transcriptomic correlation analysis.

### Generation of stable HIF knockdown cells and microarray analysis

As the Spearman correlation analysis displayed a partial compartment-specific correlation pattern and no congruent correlation over all analyzed HIF-targets, we wanted to understand the cell type-specific response to hypoxia and the relevance of given HIFs of selected renal cells. As previous studies could show that podocytes in the glomerulus and proximal tubular cells in the tubulointerstitium are the most effected cell types in the kidney^6, 16^ and to our knowledge till now no hypoxia and HIF-dependent gene expression profiles exist for either cell type, we generated proximal tubular cells (HK-2) and podocytes (AB81) with stable HIF1α and/or HIF2α suppression.

To confirm the successful suppression of both transcription factors, the cells were subjected to normoxia (N, 20% O_2_) or hypoxia (H, 1% O_2_) for 4 h or 24 h and whole cell protein extracts were immunoblotted for HIF1α and HIF2α. Immunoblot analysis revealed efficient stable HIF1α and/or HIF2α knockdown in both HK-2 cells and podocytes (Supplementary Figure 3).

To obtain a general overview of the hypoxia-response (24 h) gene expression profiles were generated. HK-2 microarrays displayed significant upregulation of 569 genes (Fold Change (FC) >1.5, q < 5%) and downregulation of 117 genes (FC < 0.667, q < 5%) under hypoxia, while in podocytes (AB81), 780 genes (FC >1.5, q < 5%) were significantly up- and 865 genes (FC < 0.667, q < 5%) significantly downregulated (Supplementary Table 2).

Microarray data analysis of these cells revealed partial cell type-specific HIF1α/HIF2α-dependencies of differentially regulated genes (Supplementary Table 2).

Quantitative RT-PCR of selected HIF-target genes was used to validate the microarray results of renal HIF knockdown cells. E.g. the well-characterized HIF-target genes carboanhydrase 9 (CA9) and VEGFA showed similar expression patterns in both cell lines. CA9 was strongly induced by hypoxia in both cells, which was abolished specifically in the absence of HIF1α. The classical HIF-target gene VEGFA displayed a moderate hypoxic induction in AB81 and HK-2 which was only lost when both HIFα isoforms were ablated. The BCL2/Adenovirus E1B 19 kDa Interacting Protein 3 (BNIP3), a well-established HIF-target gene, displayed a cell type-specific HIF1α/HIF2α dependency. While in HK-2 cells BNIP3 was HIF1α-dependently induced by hypoxia, in AB81 no hypoxic induction was observed in wildtype cells. However, in HIF2α-knockdown cells a two-fold hypoxic induction of BNIP3 could be seen. While classical HIF-target genes such as VEGFA or CA9 behaved as expected under hypoxia in podocytes, there were genes such as epidermal growth factor (EGF) and C-C motif chemokine ligand 5 (CCL5) that displayed unusual expression patterns. Expression of both genes was reduced under hypoxia compared to normoxic control. While hypoxic expression of EGF was further downregulated by ablation of HIF1α or HIF2α, CCL5 expression was decreased by HIF2α ablation, but showed a pronounced induction in HIF1α-knockdown cells. For most of the selected genes the microarray data could be confirmed by qPCR (Supplementary Table 3), excluding few genes, whose expression was either not detected with the method or showed a discrepant response to HIF-knockdown. Overall both methods were in broad agreement and consistently revealed a partial HIF1α and/or HIF2α dependency for genes in renal cell lines.

### Weighted Gene Co‑expression Network Analysis

**Figure 2:**
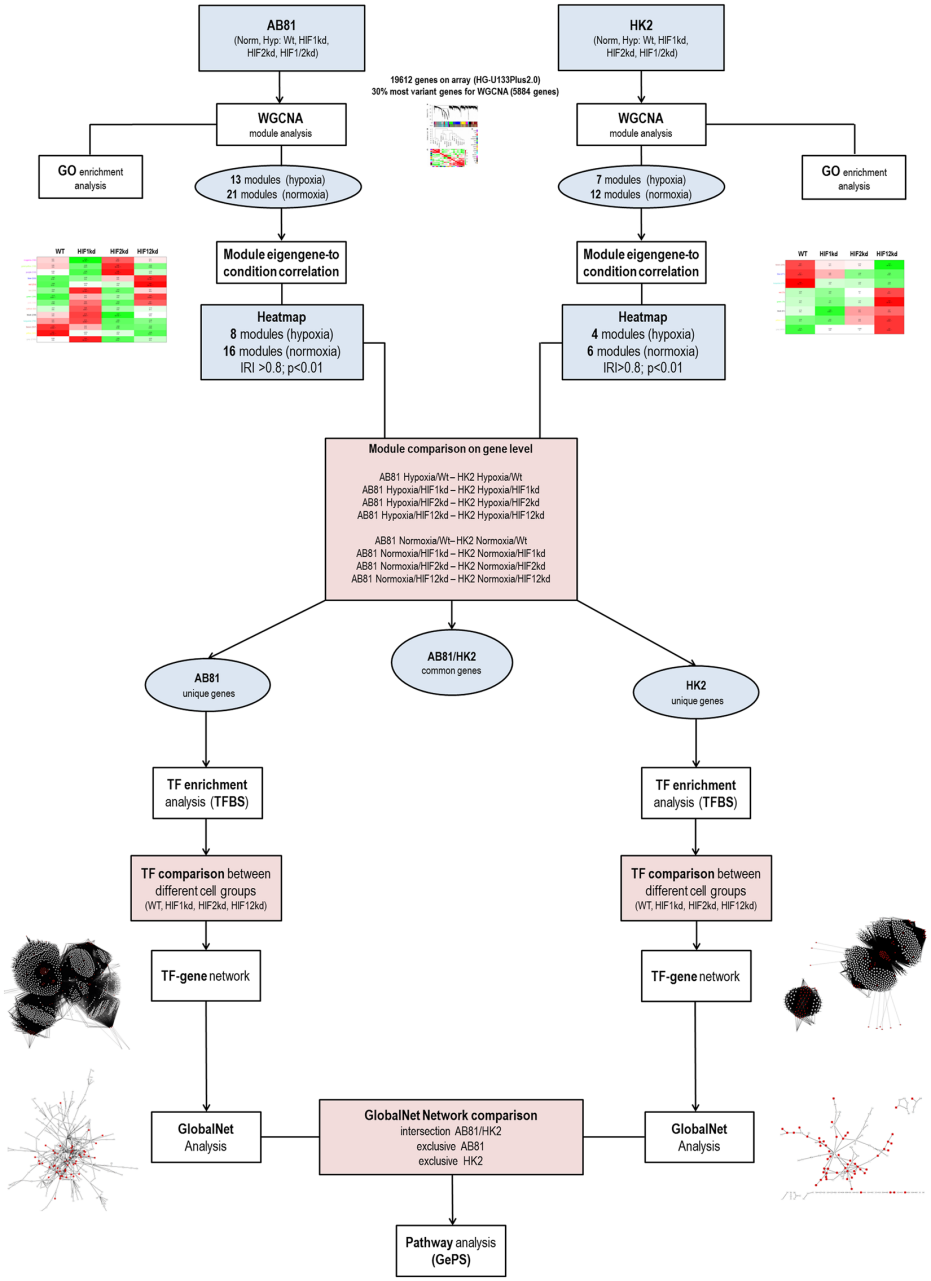
Scheme describing the workflow of microarray analysis.

To investigate hypoxia-specific gene sets associated with the respective treatment, we performed a Weighted Gene Co-expression Network Analysis (WGCNA), TF enrichment and GlobalNet analysis (Workflow, Fig. 2).

We applied the WGCNA in order to identify groups (modules) of genes that showed highly co-expressed gene expression across the four cell groups (wt, HIF1kd, HIF2kd, HIF1 + 2kd) under a specific condition (hypoxia, normoxia). As a result, for both hypoxic and normoxic conditions, we got sets of genes (modules) that were highly co-expressed within the modules, but not necessarily between the modules. We identified 34 distinct co-expression modules for AB81 [13 modules (hypoxia), 21 modules (normoxia)] and 19 modules for HK-2 [7 modules (hypoxia), 12 modules (normoxia)], respectively.

To identify gene sets correlating with HIF1α and/or HIF2α knockdown within one condition, the module eigengene (ME) corresponding to the first principal component was calculated for each module and the ME-to-condition correlations were visualized as heatmaps (Supplementary Figure 4). In podocytes all cell groups significantly correlated with at least one module under hypoxic and normoxic conditions (Supplementary Figure 4A). While under hypoxic conditions in HK-2 cells no HIF2α correlation was observed, under normoxic condition one module (red) significantly correlated with HIF2α knockdown cell group. In contrast to HIF2α, a significant correlation of modules with the HIF1α knockdown under hypoxic conditions, but no significant HIF1α correlation under normoxia was observed (Supplementary Figure 4B).

### Modules are enriched for genes with similar function

In a next step, we tried to link the module genes to biological information using a Gene Ontology (GO) analysis. Enriched GO Terms were collected across all modules for hypoxic and normoxic conditions Comparison between the different cell lines was performed resulting in six sets of GO Terms: common in hypoxia or normoxia, unique in podocytes or HK-2 cells under normoxic or hypoxic conditions (Supplementary Table 4). The GO Enrichment Analysis was combined with a network visualization of enriched GO Terms using the EnrichmentMap plugin for Cytoscape (see Materials and Methods).

**Figure 3:**
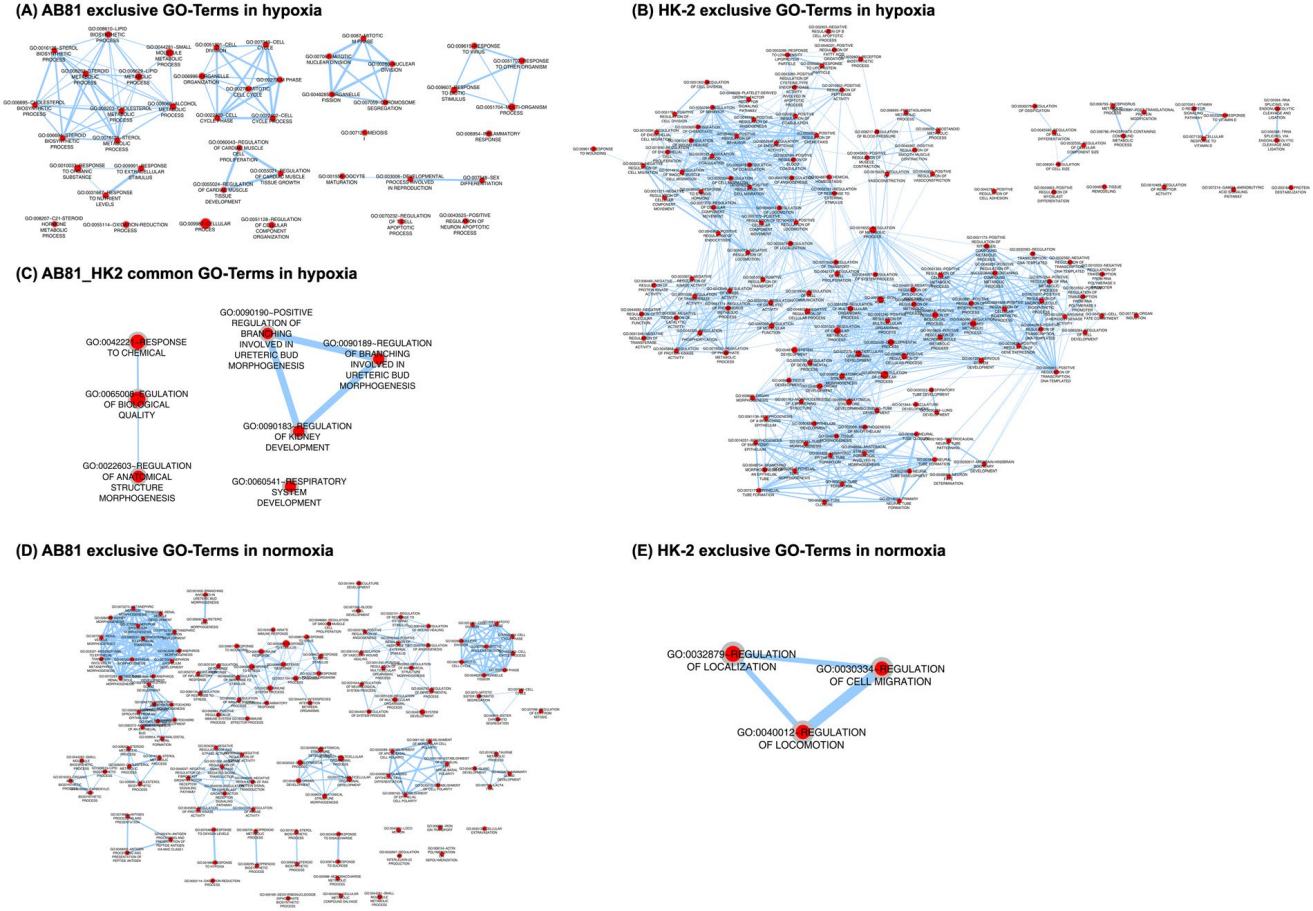
GO enrichment analysis: network visualization for the common and exclusively enriched GOs in the different cell lines using the Cytoscape plugins BinGO and EnrichmentMap. Red nodes represent enriched GO-terms, node size corresponds to negative logarithm of FDR-corrected p-value. Edge thickness shows overlap of genes between neighbor nodes. Figures (**A**) and (**B**) represent networks of GO-Terms which are exclusively associated with AB81 (**A**) and HK-2 (**B**) under hypoxic condition, figure (**C**) displays the network of common GO-Terms in AB81 and HK-2 under hypoxia. Figures (**D**–**E**) show the exlusive AB81 (**D**), HK-2 (**E**) GO-Terms under normoxic conditions. There were no common GO-Terms under normoxic conditions.

GO Term analysis revealed major differences between hypoxic treated podocytes and HK-2 cells (Fig. 3). While podocytes were mainly associated with cell cycle processes, cell differentiation, steroid/cholesterol metabolism and inflammatory responses (Fig. 3A), hypoxia induced a gene expression pattern in HK-2 cells which was associated with morphogenesis and tube formation processes (Fig. 3B), regulation of transcriptional and biosynthetic processes as well as processes involved in angiogenesis and vitamin D receptor signaling. Common processes found in both cell lines included regulation of kidney development as well as uretic bud morphogenesis (Fig. 3C). Under normoxic conditions, no common GO processes were identified between the cell lines. In podocytes, the GO network subdivided mainly into six major clusters including kidney morphogenesis, cell cycle processes, cell differentiation, steroid/cholesterol metabolism, immune responses as well as angiogenesis (Fig. 3D). HK-2 cells however were among others associated with regulation of locomotion and cell migration (Fig. 3E).

### Transcription factor (TF)-gene network

In a next step, we used the gene modules with a heatmap correlation coefficient ≥0.8 and p ≤ 0.01 for the analysis of overrepresented TF-binding sites and generation of TF-gene networks. TFs which were found to be enriched and were common among podocyte and HK-2 cell groups under hypoxic conditions are involved in cell proliferation and cell differentiation processes and belong, among others, to the “Activator protein 2” (V$AP2F), “E2F-myc activator/cell cycle regulator” (V$E2FF), “Vertebrate homologues of enhancer of split complex” (V$HESF), “Pleomorphic adenoma gene” (V$PLAG), “GC-Box factors SP1/GC” (V$SP1F) and “Krueppel like transcription factors” (V$KLFS) TF-families (Supplementary Table 5). TF-families that were found only in hypoxic podocytes and not in HK-2 cells involved families such as “Myc-interacting Zn finger protein 1” (V$MIZ1) and “CP2-erythrocyte Factor related to drosophila Elf1” (V$CP2F), which are involved in cell cycle processes or regulate steroid metabolic processes. Under normoxic conditions, no common TF-families were found in all podocyte and HK-2 cell types. However, TF-families which were enriched in most cell types included again the “E2F-myc activator/cell cycle regulator” (V$E2FF), “Pleomorphic adenoma gene” (V$PLAG), “GC-Box factors SP1/GC” (V$SP1F) and “Krueppel like transcription factors” (V$KLFS) TF-families. TF- families that were unique to normoxic podocytes involved families such as “Sterol regulatory element binding proteins” (V$SREB), V$CP2F, “Twist subfamily of class B bHLH transcription factors” (V$HAND) or “RXR heterodimer binding sites” (V$RXRF) that regulate lipid and steroid metabolic processes, angiogenesis and immune responses. TFs of the “Nuclear factor of activated T-cells” (V$NFAT) family, known to be involved in cell migration and NFAT-signaling, as well as TFs of the “Ubiquitous GLI - Krueppel like zinc finger involved in cell cycle regulation” (V$E4FF) family, known regulators of cell proliferation, were exclusively enriched in normoxic HK-2 cells (Supplementary Table 5).

**Figure 4:**
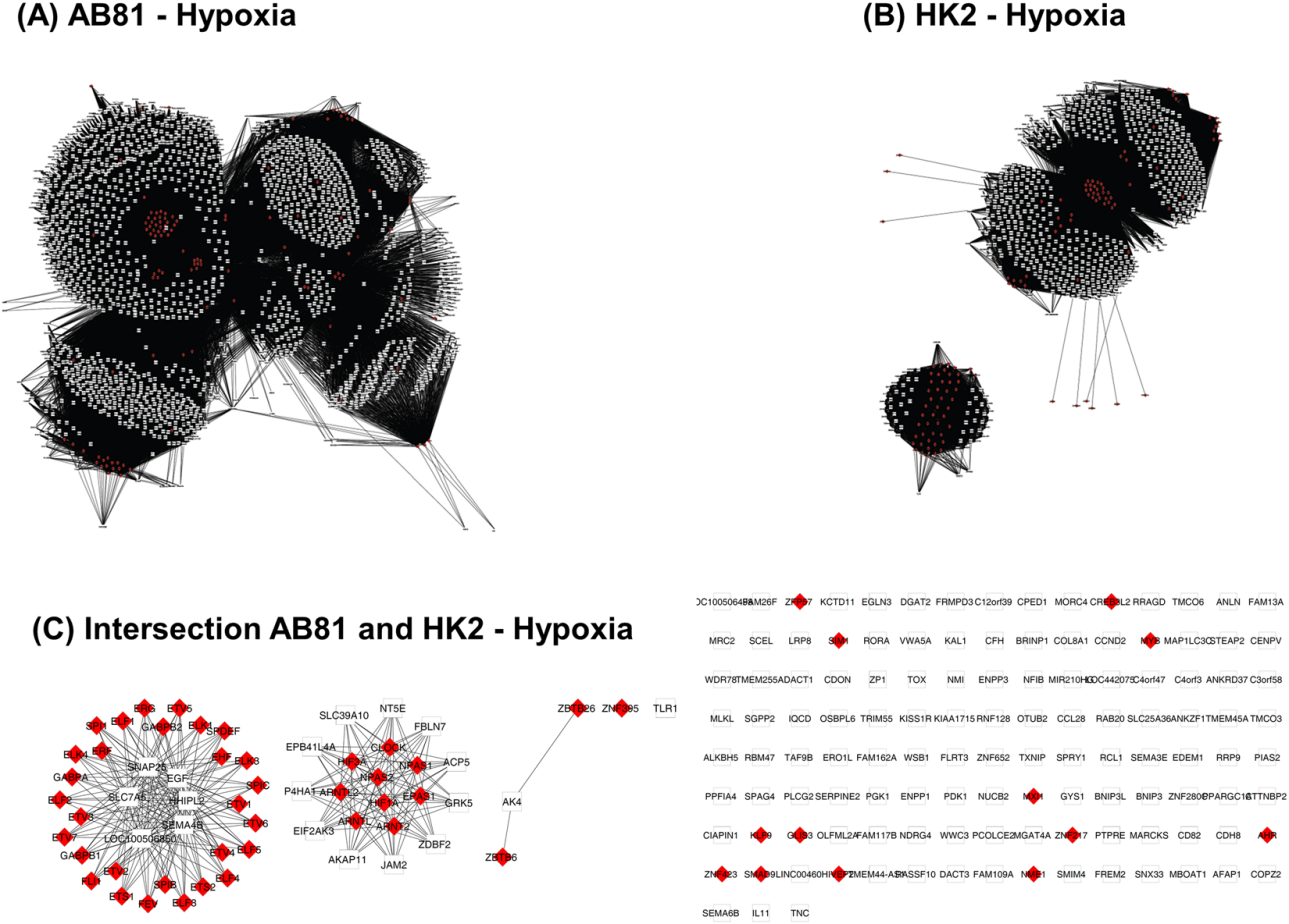
Transcription factor (TF) - gene regulatory networks (under hypoxic conditions). The podocyte TF-gene network (**A**) consists of 1837 nodes, 53175 edges. The tubular network **(B**) is compiled of 775 nodes and 24799 edges. Figure (**C**) shows the intersection of the podocyte and tubular network. Red nodes represent transcription factors, white nodes correspond to regulated genes. Edges connect TFs with their target genes.

**Figure 5:**
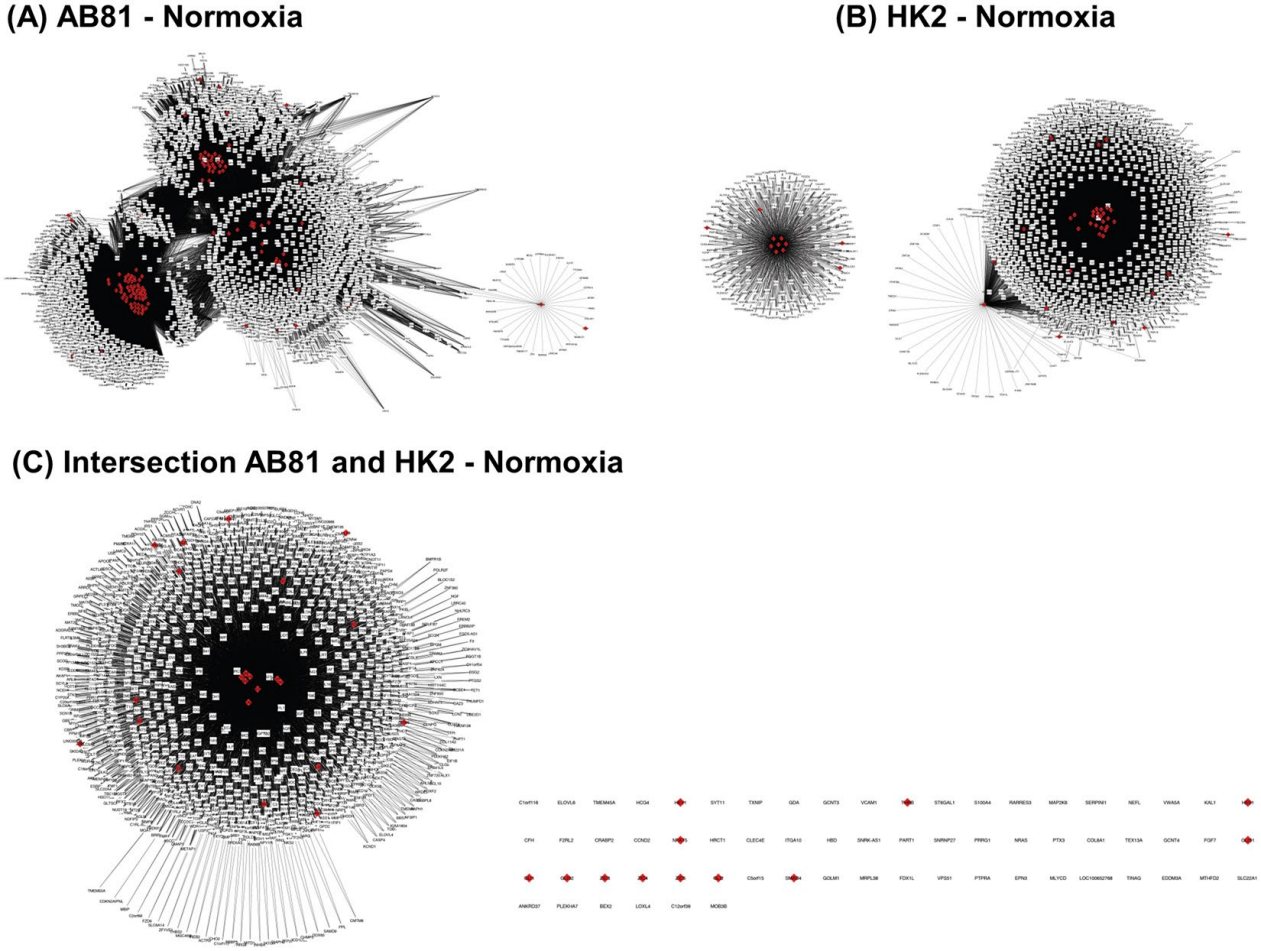
Transcription factor (TF) - gene regulatory networks (under normoxic conditions). The podocyte TF-gene network (**A**) consists of 2275 nodes, 63209 edges. The tubular network (**B**) is compiled of 1255 nodes and 21204 edges. Figure (**C**) shows the intersection of the podocyte and tubular network. Red nodes represent transcription factors, white nodes correspond to regulated genes. Edges connect TFs with their target genes.

In a next step, those single TFs were identified, which made a TF-family (identified with the Genomatix TF-enrichment analysis) being called enriched. These selected TFs were then combined with their target genes resulting in a regulatory network of enriched TFs and their regulated genes. The resulting TF-gene regulatory networks were visualized with Cytoscape (Figs 4 and 5). The hypoxia podocyte TF-gene network (Fig. 4A) consisted of 1837 nodes, 53175 edges and was denser and larger than the tubular network (Fig. 4B), which was compiled of 775 nodes and 24799 edges. Comparison of both hypoxic TF-gene networks showed a very marginal overlap between the cell types (Fig. 4C). Common TFs were mainly HIF1α and HIF2α transcription factors as well as genes from the “ETS1 factor family” (V$ETSF), which are among others involved in BMP-signaling and cellular response to oxidative stress. The normoxic TF-gene networks resulted in 2,275 nodes, 63,209 edges for the podocyte network, and 1,255 nodes, 21,204 edges for the HK-2 network (Fig. 5).

### GlobalNet and Pathway analysis

To get a more focused view on the regulatory mechanisms underlying the investigated conditions, we compared for each condition the TF-gene networks of each cell line with GlobalNet, a human protein-protein-interaction network^22^. The resulting protein-protein-interaction networks were collected for each cell line and then intersected to identify protein-protein-interaction networks that were enriched in both cell lines or were exclusively related to either cell line under hypoxic or normoxic conditions (Supplementary Figure 5). To integrate the resulting protein-protein-interaction networks into a functional context, we finally subjected these networks to a pathway analysis using the Pathway System GePS from Genomatix (Tables 2 and 3 and Supplementary Table 6). Pathways which were found in both cell lines under hypoxic conditions, included among others the “HIF1-alpha and HIF2-alpha transcription factor network” as well as the “BMP2 signaling” pathway, while the “Angiopoietin receptor Tie2-mediated signaling” and “NOTCH pathway” were among the podocyte exclusive pathways. In HK-2 cells the “EGFR1 pathway” as well as “mapkinase signaling pathway” seemed to play a major role (Table 2). Also under normoxic conditions the “BMP2 signaling” pathway seemed to be involved in both cell lines. Furthermore the “Integrin-1 pathway”, “CXCR3 pathway” and “PDGFR-beta pathway” were enriched in podocytes and HK-2 cells. Podocyte-exclusive pathways under normoxia include pathways involved in “estrogen receptor signaling”, “p38 and TGFbeta signaling” as well as the “RXR and VDR pathway”. In HK-2 cells the “Calcineurin-dependent NFAT signaling”, the “IL12-STAT4 pathway” and the “alternative NFkB pathway” were enriched (Table 3).

**Table 2:** Summary of canonical signal transduction pathways.

Canonical pathway	Pathway id	AB81_exclusive network		HK2_exclusive network		Intersection AB81_HK2	
		p-value	Adj p-value	p-value	Adj p-value	p-value	Adj p-value
Alk in cardiac myocytes	BioCarta:alkpathway	1.73E-04	0.00E+00	4.23E-04	1.00E-03		
BMP2 signaling pathway(through Smad)	INOH:BMP2_signaling_TGF-beta_MV	8.70E-03	8.00E-03	2.17E-04	0.00E+00		
Circadian rhythm pathway	NCI-nature:circadianpathway					2.30E-03	2.00E-03
HIF-1-alpha transcription factor network	NCI-nature:hif1_tfpathway	8.80E-05	0.00E+00			6.26E-04	0.00E+00
HIF-2-alpha transcription factor network	NCI-nature:hif2pathway	1.79E-03	2.00E-03	1.80E-03	2.00E-03	1.77E-04	0.00E+00
Hypoxic and oxygen homeostasis regulation of HIF-1-alpha	NCI-nature:hif1apathway			6.12E-03	1.00E-02	3.21E-03	3.00E-03
ID	CellMap:ID			3.40E-04	0.00E+00	7.95E-03	6.00E-03
Mets affect on macrophage differentiation	BioCarta:etspathway					2.73E-03	2.00E-03
Angiopoietin receptor Tie2-mediated signaling	NCI-nature:angiopoietinreceptor_pathway	1.02E-03	2.00E-03				
Estrogen responsive protein efp controls cell cycle and breast tumors growth	BioCarta:efppathway	2.33E-03	0.00E+00				
nfkb activation by nontypeable hemophilus influenzae	BioCarta:nthipathway	2.08E-03	3.00E-03				
NOTCH	CellMap:NOTCH	1.84E-03	3.00E-03				
AP-1 transcription factor network	NCI-nature:ap1_pathway			9.35E-06	0.00E+00		
EGFR1	CellMap:EGFR1			5.30E-03	1.00E-03		
Mapkinase signaling pathway	BioCarta:mapkpathway			4.92E-03	5.00E-03		
Overview of telomerase protein component gene htert transcriptional regulation	BioCarta:tertpathway			5.37E-03	7.00E-03		
Regulation of retinoblastoma protein	NCI-nature:rb_1pathway			3.57E-03	4.00E-03		
Signaling mediated by p38-alpha and p38-beta	NCI-nature:p38alphabeta-downstreampathway			1.00E-04	0.00E+00		

Adjusted p-value: corresponds to a p-value that is estimated from the results of 1,000 simulated null hypothesis queries^65^.

**Table 3:** Summary of canonical signal transduction pathways (Normoxia).

Canonical pathway	Pathway id	AB81_exclusive network		HK2_exclusive network		Intersection AB81_HK2	
		P-value	Adj p-value	P-value	Adj p-value	P-value	Adj p-value
AKT(PKB) activation signaling (Insulin receptor signaling (Mammal))	INOH:insulin_Mam					8.28E-03	6.00E-03
Beta1 integrin cell surface interactions	NCI-nature:integrin1_pathway					1.08E-03	1.00E-03
BMP receptor signaling	NCI-nature:bmppathway					2.18E-03	1.00E-03
BMP2 signaling pathway(through TAK1)	INOH:BMP2_signaling_TAK1	1.68E-03	2.00E-03			8.61E-03	9.00E-03
CXCR3-mediated signaling events	NCI-nature:cxcr3pathway	4.29E-04	0.00E + 00			4.27E-03	3.00E-03
human cytomegalovirus and map kinase pathways	BioCarta:hcmvpathway					2.05E-03	0.00E + 00
PDGFR-beta signaling pathway	NCI-nature:pdgfrbpathway	3.12E-03	2.00E-03	1.54E-03	2.00E-03		
A6b1 and a6b4 Integrin signaling	NCI-nature:a6b1_a6b4_integrin_pathway	6.88E-03	4.00E-03				
Aspirin blocks signaling pathway involved in platelet activation	BioCarta:sppapathway	3.52E-03	4.00E-03				
ATF-2 transcription factor network	NCI-nature:atf2_pathway	5.16E-03	7.00E-03				
Aurora B signaling	NCI-nature:aurora_b_pathway	9.19E-03	5.00E-03				
Ctcf: first multivalent nuclear factor	BioCarta:ctcfpathway	4.21E-03	5.00E-03				
EGF receptor proximal signaling	NCI-nature:erbb1_receptor_proximal_pathway	2.70E-03	2.00E-03				
EPHB forward signaling	NCI-nature:ephbfwdpathway	2.20E-03	3.00E-03				
Internalization of ErbB1	NCI-nature:erbb1_internalization_pathway	4.30E-03	5.00E-03				
Nfkb activation by nontypeable hemophilus influenzae	BioCarta:nthipathway	1.33E-03	1.00E-03				
p38 cascade (TGF-beta signaling(through TAK1))	INOH:TGF-beta_signaling_TAK1	1.71E-03	3.00E-03				
p38 signaling mediated by MAPKAP kinases	NCI-nature:p38_mk2pathway	2.36E-03	2.00E-03				
Phosphorylation of mek1 by cdk5/p35 down regulates the map kinase pathway	BioCarta:cdk5pathway	7.31E-03	6.00E-03				
Plasma membrane estrogen receptor signaling	NCI-nature:er_nongenomic_pathway	8.93E-04	0.00E + 00				
Ras signaling in the CD4 + TCR pathway	NCI-nature:tcrraspathway	7.31E-03	3.00E-03				
RXR and RAR heterodimerization with other nuclear receptor	NCI-nature:rxr_vdr_pathway	1.39E-04	0.00E + 00				
Signaling mediated by p38-alpha and p38-beta	NCI-nature:p38alphabetadownstreampathway	7.69E-03	8.00E-03				
Tgf beta signaling pathway	BioCarta:tgfbpathway	8.63E-03	8.00E-03				
Trk receptor signaling mediated by PI3K and PLC-gamma	NCI-nature:pi3kplctrkpathway	1.40E-03	1.00E-03				
Alpha-synuclein signaling	NCI-nature:alphasynuclein_pathway			6.14E-03	5.00E-03		
Alternative NF-kappaB pathway	NCI-nature:nfkappabalternativepathway			7.67E-03	7.00E-03		
Amb2 Integrin signaling	NCI-nature:amb2_neutrophils_pathway			5.82E-03	5.00E-03		
Calcium signaling in the CD4 + TCR pathway	NCI-nature:tcrcalciumpathway			4.85E-03	3.00E-03		
FoxO family signaling	NCI-nature:foxopathway			6.34E-03	5.00E-03		
IL12 signaling mediated by STAT4	NCI-nature:il12_stat4pathway			1.17E-03	2.00E-03		
Role of Calcineurin-dependent NFAT signaling in lymphocytes	NCI-nature:nfat_3pathway			1.32E-03	1.00E-03		
Validated transcriptional targets of AP1 family members Fra1 and Fra2	NCI-nature:fra_pathway			9.37E-03	9.00E-03		

Adjusted p-value: corresponds to a p-value that is estimated from the results of 1,000 simulated null hypothesis queries^65^.

**Figure 6:**
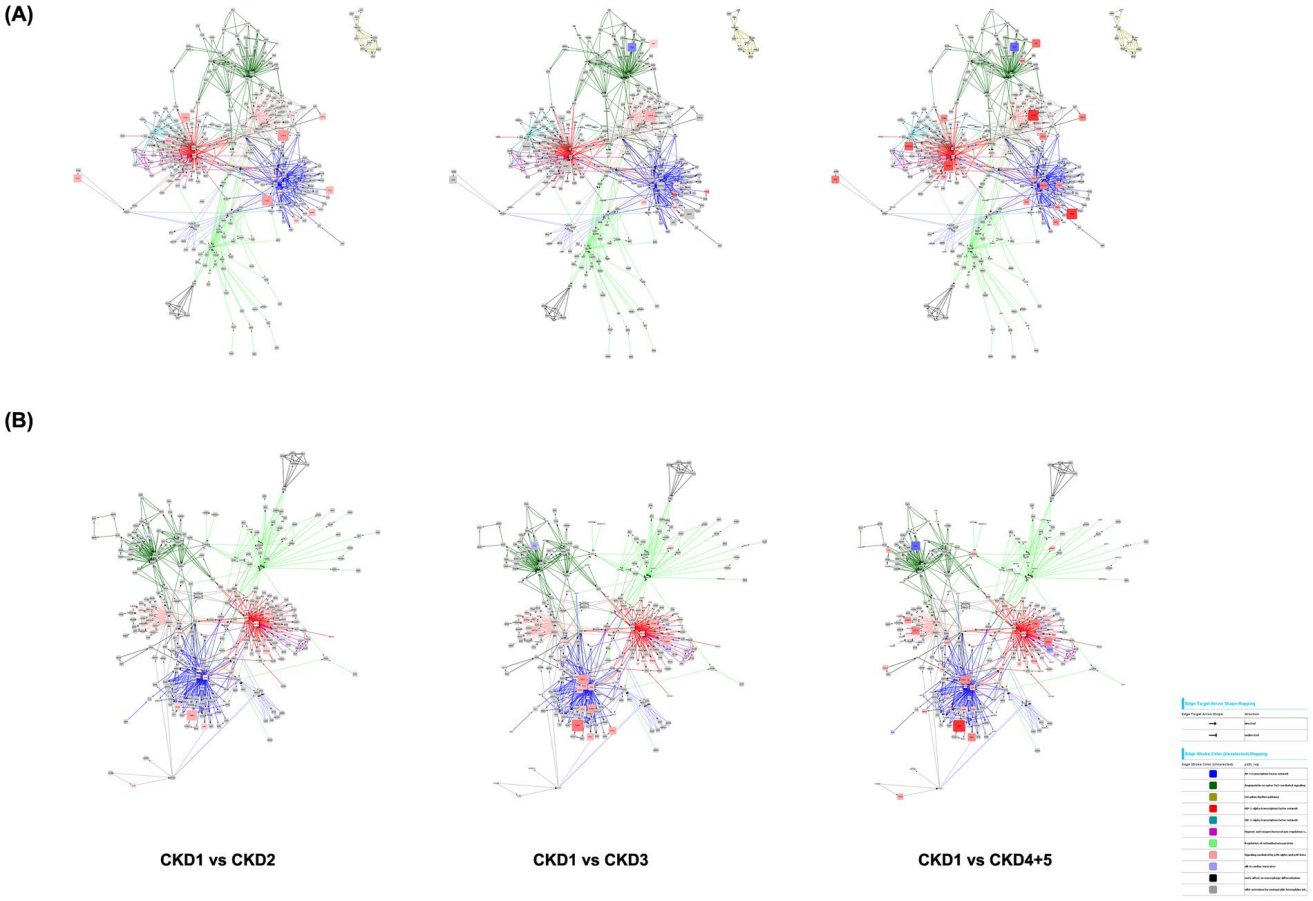
Visualization of the interplay of the significantly enriched pathways in CKD development progression. Increased dysregulation of the hypoxia-interconnected pathways with loss of renal function in glomerular (**A**) and tubulointerstitial (**B**) samples. Color represents the log2 expression foldchange, node size the absolute value of the log2 foldchange, edge color represents the pathway. Directionality is based on pathway information and represented by arrows. Red means upregulated, blue downregulated compared to CKD1.

Many of the identified biological pathways are described to be involved in CKD development and progression. Analysis of the gene expression of the pathway members could confirm the dysregulation of the hypoxia-interconnected pathways in glomeruli and tubulointerstitium in CKD (Supplementary Table 7) and revealed an increased dysregulation of these pathways with disease progression (Figure 6). Gender-specific analysis showed mainly differences in early stages, while with progression of the disease dysregulation of the pathways were similar in both sexes (Supplementary Table 7).

## Discussion

CKD strongly influences whole body homeostasis. With progressive loss of renal function, patients with CKD develop beside anemia disturbances in electrolyte, water and acid-base homeostasis, as well as rapid atherosclerosis and osteopathy. Previous studies could demonstrate that oxygen sensing and hypoxia-associated gene regulation seem to be central mechanisms in the developing, mature and diseased kidney^23^. Different detection methods such as O_2_ microelectrodes, pimonidazole staining or transgenic mice have been used to reveal renal hypoxia *in vivo* in different animal models, including glomerulonephritis, polycystic kidney disease, unilateral ureteral obstruction (UUO) and the aging kidney^6, 24^. Due to this accumulating evidence Fine and Norman^6^ suggested the “chronic hypoxia hypothesis”, where hypoxia plays a key role in loss of renal function and development of CKD. However, human data supporting the “chronic hypoxia hypothesis” are difficult to generate and are therefore limited. In this study, we assessed the expression of hypoxia-associated genes in genome-wide expression profiles of more than 200 renal biopsies from patients with different CKD stages which revealed significant correlation of several HIF-target genes with eGFR in the glomerular samples and cortical tubulointerstitium. These correlations were both positive and negative and in part compartment-specific. Our data clearly point to a compartment-specific dysregulation of hypoxia-associated gene transcripts. While the observed enrichment for hypoxia-associated genes being correlated with eGFR is very unlikely to have occurred just by chance for glomerular samples, the eGFR correlation with tubulointerstitial expression patterns does not seem to be specific for hypoxia-associated genes as shown by the bootstrapping analysis. Although the expression of HIF-target genes is only an indirect measure for hypoxia, our data do not seem to support the hypothesis of an overall hypoxic milieu in the diseased kidney. In line with these results are BOLD-MRI studies in CKD patients. While some studies showed a decrease in oxygen tension in the human kidneys with CKD using BOLD-MRI^18, 25–27^, others could not demonstrate a loss of oxygenation in kidneys of different degrees of kidney function^28, 29^. However, we cannot exclude that environmental factors in the CKD milieu, such as uremia or oxidative stress, might modulate HIF function and thereby influence the hypoxic response and the expression of respective HIF target genes. A recent study by Tanaka *et al*. could demonstrate that indoxyl sulfate, a uremic toxin, impaired the hypoxic response in proximal tubular cells and in experimental CKD by mRNA stabilization of CITED2. Elimination of indoxyl sulfate by AST-120 restored the hypoxic expression of HIF target genes^30^. In recent years several lines of evidence suggest hypoxia as a powerful stimulus regulating the expression of selective miRNAs. These hypoxia-induced miRNAs play important roles in metabolism, cell cycle progression, apoptosis or angiogenesis^31^. However, the roles of a miRNA can vary depending on the setting. E.g. miR-21, a miRNA involved in renal fibrosis and the pathogenesis of ischemia/reperfusion injury, seems to regulate both protective and pathological pathways. While preconditioning-induced upregulation of miR-21 contributes to the protection against subsequent renal I/R injury, long-term elevation of miR-21 seems to be detrimental to the organ by promoting the development of renal interstitial fibrosis following I/R injury^32^.

As we found in part a compartment-specific regulation of selected HIF-target genes, we analyzed the impact of HIF1α and/or HIF2α in two renal cell lines – AB81, an immortalized human podocyte cell line, and HK-2, a human proximal tubular cell line. We chose these cell lines, as in previous studies we could show that podocytes in the glomerulus^16^ and proximal tubular cells in the tubulointerstitium^15^ seem to be the prominently effected cells in kidneys of patients with nephrosclerosis or diabetic nephropathy (DN). To assess the transcriptional impact of HIFs in the different cell lines genome-wide gene expression profiles of stable HIF1α and/or HIF2α knockdowns from each cell line under normoxic and hypoxic conditions have been generated and analyzed. The podocyte cell line seemed to be more susceptible to hypoxia than HK-2 cells, as a higher fraction of genes were significantly dysregulated under hypoxic condition. Interestingly, this would be in accordance with the eGFR correlation and bootstrapping analysis results.

To understand the biological context behind the transcriptional regulation in the different cell lines, we investigated hypoxia-specific gene sets associated with the respective activation program, applying WGCNA which identifies groups of genes that showed highly correlated gene expression across four cell groups (wt, HIF1kd, HIF2kd, HIF1 + 2kd) under hypoxic or normoxic conditions. Combination with a GO enrichment analysis, TF-gene network and pathway analysis allowed us to distinguish between common and specific biological processes and regulatory mechanisms between the two cell lines.

Biological processes which have been common in podocytes and HK-2 cells under hypoxic conditions involved kidney development and uretic bud morphogenesis. This is in accordance with previous studies, which could show that during development kidneys are exposed to hypoxia^33^. A recent study could demonstrate that hypoxia significantly reduced the size of metanephric kidneys, as well as the number of branches and glomeruli at development^34^. Along with these studies is our enrichment analysis of the HIF1α and HIF2α transcription factor network in both cells. Additionally, we identified “BMP2-signaling pathway” being influenced in both cell lines under hypoxic conditions. Bone morphogenic proteins (BMPs) belong to the TGF-β superfamily and have been shown to regulate among others kidney development and the pattern of the uretic tree^35^. Perturbations in BMP-signaling pathways have been linked to developmental disorders as well as to the pathophysiology of several diseases, including renal fibrosis and kidney diseases. E.g. BMP7 has been described as being protective to adult nephrons and to have strong anti-fibrotic activity by counteracting the effects of TGFβ1^36, 37^. Our gene expression analysis of different CKD stages showed a significant loss of BMP7 with loss of renal function, while TGFβ1 was significantly upregulated (Supplementary Table 7, Fig. 6), indicating a shift towards profibrotic activity with CKD progression. BMPs as well as TGFβ transduce their signals through SMAD and non-SMAD signaling pathways. Recent studies showed an interaction of HIF1α with SMAD3, a downstream transcription factor of TGFβ signaling, supporting a possible role in kidney injury and glomerulosclerosis, by activation of profibrotic genes such as COL1A1, 2 or SERPINE1^38, 39^.

The podocyte is a highly-specialized cell type with unique structure. One characteristic of mature podocytes is their terminal differentiation status and thereby their lack of proliferation. However, under certain circumstances such as collapsing focal segmental glomerulosclerosis podocytes seem to be able to reenter the cell cycle, leading to cell proliferation^40, 41^, mitotic catastrophe with subsequent cell death and glomerulosclerosis^42^. Notch, which is normally not expressed in healthy glomeruli, has been identified as one important regulator for the re-entrance into the cell cycle^43, 44^. In this study, among the identified podocyte-specific processes and pathways cell cycle and cell division processes as well as the Notch pathway have been specified, indicating a crosslink of hypoxia to Notch and cell cycle deregulation in the podocytes. From the module coexpression analysis we found an association of these podocyte-specific processes with HIF1α and/or HIF2α. A study by Gustaffson and colleagues could demonstrate, that hypoxia activates Notch signaling due to the association of Notch and HIF1α thereby negatively blocking cell differentiation^45^.

Another major activated/impaired GO-subcluster in the podocyte cells that has been identified in this study is the steroid/cholesterol metabolism. This corresponds with several studies which could show that lipids and cholesterol are important for the podocyte structure and function, so e.g. cholesterol is required for the localization and function of the slit-diaphragm proteins in the lipid rafts^46^. Recently, it has been demonstrated that an imbalance in the cholesterol homeostasis due to increased synthesis, influx or decreased efflux is associated with the development of proteinuric diseases such as minimal change disease, focal segmental glomerulosclerosis (FSGS) or DN^47^, e.g. patients with mutations in genes of the cholesterol metabolism such as APOL1 or APOE were shown to be more susceptible to FSGS (APOL1) or develop a lipoprotein glomerulopathy (APOE), while patients with DN showed a reduced expression of ABAC1, indicating that lipid accumulation in podocytes might be due to a defective cellular cholesterol homeostasis^47^.

In the tested HK-2 cells major regulated gene clusters were related to morphogenesis/tube formation and regulation of angiogenesis. Previous studies have shown that VEGFA, which is mainly expressed by podocytes and proximal tubular cells, induces capillary formation by endothelial cells and that hypoxia augments branching angiogenesis^48^. CKD is often associated with rarefaction of peritubular capillaries, leading to a decrease in oxygen supply, finally resulting in hypoxia. In advanced stages of CKD VEGFA expression is reduced^15, 17^. In accordance with the regulation of angiogenesis and morphogenesis in hypoxic HK-2 cells is, besides BMP2 signaling, the enrichment for the AP1 transcription factor network and EGFR1-signaling pathway, which are both important pathways that regulate growth, survival, proliferation, and differentiation. A study by Kolev *et al*. could show that EGFR signaling is a negative regulator of Notch1, promoting cell proliferation and that knockdown of c-Jun and c-Fos, downstream effectors of EGFR, lead to an induction of Notch1^49^. In our study, most processes of these clusters were related to modules which correlated mainly to wild type HK-2 cells, indicating that hypoxia-inducible factors do not play a major role in these processes.

Most of the hypoxia associated biological processes in the podocytes were also found in the normoxic analysis indicating a HIF1 and/or HIF2-dependencies of these processes. In HK-2 cells, however, only processes involved in cell migration seemed to be common under both normoxic and hypoxic conditions, while most of the observed hypoxia associated processes (at 24 h) did not show evidence for direct HIF-dependency.

While HIF1α is preferentially involved in glycolytic pathways, HIF2α is among others involved in the regulation of angiogenesis, cell cycle progression and cell proliferation^50^, which is in accordance with the finding that podocyte undergo cell cycle dysregulation under hypoxic conditions and are known to express HIF2α. When we intersected the TF-gene networks of the two cell lines, HK-2 and podocytes, we found a very small overlap, mostly involving HIF transcription factors and factors of the ETS-family. ETS transcription factors have been shown to cooperate specifically with HIF2α (EPO, VEGFR2, CITED2) and thereby promote angiogenesis^51^.

In conclusion, our data clearly point to a compartment- and cell type-specific dysregulation of hypoxia-associated gene transcripts. Co-expression and network analysis of HIF-knockdown cells identified biological processes and pathways known to be involved in the development of CKD. Comparison of these hypoxia-interconnected pathways with gene expression data of patients with different CKD stages revealed an increased dysregulation of the pathways with growing loss of renal function. With currently several clinical trials of novel PHD enzyme inhibitors for the treatment of renal anemia being performed, a better understanding of the renal oxygen signaling pathway becomes even more important. Our dataset might thereby help to obtain an improved understanding of the renal effects of hypoxia, HIF dysregulation and the transcriptional program response in CKD.

## Material and Methods

### Patient characteristics

Human renal biopsy specimens and Affymetrix microarray expression data were procured within the framework of the European Renal cDNA Bank - Kröner-Fresenius Biopsy Bank^52, 53^. Biopsies were obtained from patients after informed consent and with approval of the local ethics committees (Specialized Ethics Subcommittee of Internal Medicine of the University Hospital Zurich, Cantonal Ethics Committee Zurich and Ethics Committee of the Medical Faculty of the University of Munich (LMU)). All methods were performed in accordance with the relevant guidelines and regulations. Histology reports, clinical data, and gene expression information were stored in a de-identified manner. A total of 362 microarrays from kidney biopsies of 201 patients were used for gene expression analysis and kidney function correlation analysis (GSE47185, GSE32591, GSE37463). Demographic data of these 194 patients are provided in Supplementary Table 8.

### RNA Isolation, RNA Preparation, and Microarray Data Analyses of microdissected human kidney biopsies

Following renal biopsy, the tissue was transferred to RNase inhibitor and microdissected into glomerular and tubulointerstitial fragments. Total RNA was isolated from microdissected glomeruli and tubulointerstitium, reverse transcribed, and linearly amplified according to a protocol previously reported^54^. Affymetrix GeneChip Human Genome U133A and U133 Plus2.0 Arrays were used in this study. The microarray expression data came from individual patients with different chronic kidney diseases (cadaveric donor (CD), tumor nephrectomy (TN), diabetic nephropathy (DN), thin basement disease (TMD), minimal change disease (MCD), hypertensive nephropathy (HTN), IgA nephropathy (IgA), focal segmental glomerulosclerosis (FSGS), membranous nephropathy (MGN), lupus nephritis (LN) and ANCA-vasculitis (ANCA)). Fragmentation, hybridization, staining, and imaging were performed according to the Affymetrix Expression Analysis Technical Manual (Affymetrix, Santa Clara, CA). The raw data was normalized using Robust Multichip Algorithm (RMA) and annotated by Human Entrez Gene custom CDF annotation version 18 (http://brainarray.mbni.med.umich.edu/Brainarray/default.asp). The log transformed dataset was corrected for batch effect using ComBat from the GenePattern pipeline (http://www.broadinstitute.org/cancer/software/genepattern/)^55^. Normalized data are available at the Gene Expression Omnibus (GEO) Web site (http://www.ncbi.nlm.nih.gov/geo/) under accession number GSE99340.

### Correlation of gene expression data with clinical parameters and differential gene expression analysis

To analyze the overrepresentation of HIF-induced genes in CKD, we compiled a list of well-known HIF target genes from the literature. Only HIF targets in which binding of HIF to the target DNA sequence in a DNA binding assay, a functional transactivation of reporter gene expression or a knock-down experiment has been shown were selected (resulting in a list of 84 HIF-target genes, Supplementary Table 9)^20, 21^. Except of one gene (LDHA) all genes were found on the Affymetrix microarray HG-U133A. We refer to these 83 genes as HIF target genes. Tubulointerstitial and glomerular gene expression profiles from patients with different chronic kidney diseases (cadaveric donor (CD), tumor nephrectomy (TN), diabetic nephropathy (DN), thin basement disease (TMD), minimal change disease (MCD), hypertensive nephropathy (HTN), IgA nephropathy (IgA), focal segmental glomerulosclerosis (FSGS), membranous nephropathy (MGN), lupus nephritis (LN) and ANCA-vasculitis (ANCA)) were used to compute the correlation of estimated GFR (eGFR) calculated by the CKD Epidemiology Collaboration (CKD-EPI) equation^56^ with the log-transformed steady-state expression levels of the HIF target genes within each subject using Spearman correlation. Disease cohort sizes ranged from 3 to 30 patients, with LN (30 patients) and IgA (26 patients) being the largest subcohorts. To assess the effect of demographic factors such as gender, correlation analyses were performed separately for females and males.

For the differential gene expression analysis the patients were grouped into different CKD stages (CKD1-5) according to their estimated GFR calculated by the CKD-EPI equation^57^ (Glom: CKD1: n = 55; CKD2: n = 52; CKD3: n = 44; CKD4: n = 26; CKD5: n = 10; Tub: CKD1: n = 56; CKD2: n = 46; CKD3: n = 37; CKD4: n = 26; CKD5: n = 10), where the CKD1 group served as the control group. To identify differentially expressed genes the SAM (Significance analysis of Microarrays) method was applied using TiGR (MeV, Version 4.8.1)^58^. A q-value below 5% was considered to be statistically significant. Additionally, we assessed to what extent gene expression changes were observed in both sexes by performing the differential gene expression analysis separately for females and males.

### Immunohistochemistry on biopsies for selected HIF target genes/Tissue array

Sections from paraffin embedded kidney biopsies with chronic kidney disease (normal and reduced eGFR) and kidney tissue obtained from tumor nephrectomy specimens (n = 54) were stained with antibodies against HIF1α, VEGFA, and ABCG2. After antigen retrieval, slides were incubated with the following antibodies: HIF1α clone mgc3 ab16066 (Abcam, Cambridge, UK), dilution 1:400; VEGFA sc-152 (Santa Cruz Biotechnology), dilution 1:300; ABCG2 ab3380 (Abcam, Cambridge, UK), dilution 1:60. The immunohistochemical staining was conducted on automated staining systems (ABCG2 on Ventana BenchMark, Ventana Medical Systems, USA; HIF1α and VEGFA on Leica Bond System, Leica Biosystems) following the manufacturer’s instructions. Visualization was performed using the avidin–biotin complex method leading to a brown staining signal. For all stainings the intensity in the glomerular and tubulointerstitial compartment (negative = score 0, weak = score 1, strong = score 2) in comparison to expression in normal tissue was assessed. Statistical analysis using Fisher’s exact test was performed with SPSS statistics software version 22 (IBM, USA). p-values < 0.05 were considered as significant.

### Cell Culture

Immortalized human podocytes (AB81) and human proximal tubular epithelial cells (HK-2, (ATCC CRL-2190)) were cultured as described before^16, 59^. Briefly, conditionally immortalized human podocytes were cultured under permissive conditions (33 °C) in RPMI-1640 supplemented with 10% FBS, penicillin (100 U/ml)/streptomycin (100 µg/ml), and 1% ITS. Differentiation was induced by thermoshift to 37 °C in 6-well plates (RNA isolation) and 15 cm dishes (protein isolation). HK-2 cells were cultured in DMEM/F-12 supplemented with 10% fetal calf serum (FCS), 1% ITS, hydrocortisone (36 ng/ml), penicillin (100 U/ml) and streptomycin (100 µg/ml). The cells were incubated at 37 °C.

Normoxia was defined as 20% O_2_ in the gas phase and hypoxia constituted 1.0% O_2_, respectively. For oxygen titration, cell cultures were distributed into incubators (Binder, Tuttlingen, Germany) with different oxygen concentrations (1.0% and 20% O_2_) and simultaneously cultured for the respective time. Cells were harvested and analyzed for HIF1α and HIF2α expression by Western blot and mRNA expression analysis, respectively. Total cellular RNA was extracted using the Qiagen RNeasy kit (Qiagen, Hombrechtikon, Switzerland). mRNA levels were analyzed by real-time RT-PCR and microarray experiments.

### HIFα knock-down cells and lentiviral infections

shHIF1A knock-down cells were generated by transfection with a pLKO.1-puro vector expressing U6 promoter driven shRNA targeting nucleotides 1168–1188 of human HIF1A (NM_001530.x-1048s1c1, Sigma). Cell clones were derived by puromycin selection (8 μg/ml) and ring cloning. For shHIF2A knock-down cells a lentiviral expression vector encoding a shRNA sequence targeting human HIF2A at nucleotides 2055–2075 (NM_001430.x-1694s1c1) in a pLKO.1-puro plasmid was purchased from Sigma. Variants of shRNA expression plasmids bearing a blasticidin resistance marker were generated by replacing the original puromycin resistance cassette in pLKO.1. Viral particles were produced in HEK293T cells by co-transfection of the respective transfer vector (3 μg) with the packaging plasmids pLP1 (4.2 μg), pLP2 (2 μg) and pVSV-G (2.8 μg, all from Invitrogen) using PEI transfection as described before^57^. Podocytes and HK-2 cells were infected with lentiviral-pseudotyped particles and cell pools were derived by puromycin (8 μg/ml) and/or blasticidin (20 μg/ml) selection.

### Western Blot analysis

For HIF1α detection, cultured cells were harvested with lysis buffer (10 mM Tris-HCl [pH 8.0], 0.1% Nonidet P-40, 400 mM NaCl, 1 mM EDTA [pH 8.0], and 1 mM DTT, 1 mM PMSF and Complete Protease Inhibitor Cocktail (Roche, Mannheim, Germany)). For HIF2α detection, cells were harvested with a lysis buffer containing 8 M urea (10 mM Tris-HCl [pH 6.8], 1% SDS, 5 mM DTT, 10% Glycerol, 8 M Urea, 0.5 mM PMSF and Complete Protease Inhibitor Cocktail (Roche, Mannheim, Germany)). The protein concentrations were determined by the Bradford method (Bio-Rad). Extracted proteins were boiled in loading buffer for 5 min, resolved by 10% SDS-PAGE under reducing conditions, and transferred to an Immobilon-P membrane (Millipore, Eschborn, Germany). Equal loading and transfer efficiency were verified by staining with 2% Ponceau S. Membranes were blocked overnight with Tris-buffered saline (TBS) / 5% fat-free skim milk and then incubated with a monoclonal mouse antibody raised against human HIF1α (BD Transduction Laboratories, Lexington, KY) diluted 1:1000 overnight at 4 °C, and rinsed with TBS that contained 0.1% Tween 20. HIF2α was detected using a polyclonal rabbit anti-HIF2α antibody (1:200, overnight at 4 °C; Novus Biologicals, Littleton, CO, USA).

For detection, a horseradish peroxidase–linked anti–mouse IgG antibody (1:2000, 1 hour at room temperature; DAKO, Glostrup, Denmark) and enhanced chemiluminescence substrate (PerkinElmer) were used. Membranes were also probed with anti-beta-actin antibody (A 5316, 1:5000, Sigma-Aldrich, Germany) as internal loading control.

### Microarray analysis of HIFα knock-down cells

For oxygen titration, cell cultures were distributed into incubators (Binder, Tuttlingen, Germany) with different oxygen concentrations (1.0% and 20% O2) and simultaneously cultured for 24 hours. Total cellular RNA was extracted using the Qiagen RNeasy kit (Qiagen, Hombrechtikon, Switzerland). For RNA amplification which was performed with the Ovation^TM^ RNA Amplification System V2 (NuGen Inc), 50 ng of total RNA was used. The amplified cDNA was purified according to the manual instruction using Agentcourt Beads RNA Clean (Agencourt – Beckman Coulter Genomics). For fragmentation and biotin labelling 3.3 µg of each amplified cDNA was processed with the NuGen Encore Biotin Module (NuGen Inc.). All sample processing procedures were performed according to the user manuals. Quality control of all steps was determined by the Agilent Bioanalyzer. cDNA was hybridized to Affymetrix GeneChip Human Genome U133Plus2.0. Hybridization, staining, and imaging were performed according to the Affymetrix Expression Analysis Technical Manual (Affymetrix, Santa Clara, CA). The raw data was normalized using Robust Multichip Algorithm (RMA) and annotated by Human Entrez Gene custom CDF annotation version 18 (http://brainarray.mbni.med.umich.edu/Brainarray/default.asp). For HK-2 data, a batch correction using ComBat from the GenePattern pipeline (http://www.broadinstitute.org/cancer/software/genepattern/) was applied^55^, as these data originated from different batches. To identify differentially expressed genes the SAM (Significance analysis of Microarrays) method was applied using TiGR (MeV, Version 4.8.1)^58^. A q-value below 5% was considered to be statistically significant. Normalized data are available at the Gene Expression Omnibus (GEO) Web site (http://www.ncbi.nlm.nih.gov/geo/) under accession number GSE99340.

### Weighted Gene Coexpression Network Analysis (WGCNA)

Prior to WGCNA, the gene expression data were pre-processed with RMA. For HK-2 data, a batch correction using ComBat from the GenePattern pipeline (http://www.broadinstitute.org/cancer/software/genepattern/) was applied^55^, as these data originated from different batches, in contrast to AB81 data. Individual weighted gene co-expression network analyses (WGCNA)^60^ were performed individually for AB81 and HK-2 cells, respectively, using WGCNA for R, version 1.41-1. For both cell types, two analyses were done: one with sub-division for knockdown type (wt, HIF1kd, HIF2kd, HIF1 + 2kd) and the other with respect to hypoxic/normoxic conditions. Each WGCNA was performed as follows. First, the 30% of genes being most variable in their gene expression across samples of the same type (e.g. HIF1kd) were selected for further analysis. Genes and samples were then tested for too many missing values with the method goodSamplesGenes of WGCNA package for R. Search for outlying samples was performed according to WGCNA default settings. None of the samples had to be removed. The soft threshold (power value), which is critical for WGCNA, was determined according to WGCNA tutorial recommendation by choosing the lowest power value which gives an R^2 of at least 0.7 for the scale free topology model fit. The remaining WGCNA parameters were set as follows: mergeCutHeight 0.05 for Hypoxia/Normoxia and 0.3 for knockdown type analyses, respectively, minModuleSize 50, minCoreKME 0.9 and minKMEtoStay 0.85. KME describes the correlation of a gene’s expression profile with that of the first principal component of the expression matrix. The first principal component is called a module eigengene (ME). The KME value can thus be interpreted as a module membership value for a gene and a WGCNA module. The MEs were calculated for all modules and a ME-to-treat (condition) correlation was determined which was visualized as a heatmap. To distinguish one module from another, each module was assigned a color, with grey being reserved for the unassigned genes.

### GO Term Enrichment Analysis

For the gene modules originating from WGCNA, individual GO Term Enrichment Analyses were performed with the Cytoscape plugin BinGO (BinGO Version 3.03; Cytoscape Version 3.2.1)^61^. GO Terms were called significant when their Benjamini-Hochberg-corrected p-value (FDR) for enrichment was below 0.05. The significant GO Terms were collected across all modules of the same WGCNA and were then compared against the significant GO Terms from the respective WGCNA (e.g. the GO Terms of AB81 under hypoxic conditions were compared with those of AB81 under normoxic conditions). These comparisons gave GO Terms that were common between (e.g. hypoxic and normoxic) conditions and also GO Terms that were exclusively enriched in only one of both conditions. Visualization of GO-enrichment was performed using the Cytoscape plugin EnrichmentMap (EnrichmentMap v2.0.1)^62^.

### TF Enrichment Analysis with Genomatix

WGCNA-based gene modules were used as gene sets for the overrepresented transcription factor binding sites (OTFBS) analysis (Genomatix). From the hypoxia and normoxia experiments in each cell line (AB81 and HK-2) only gene modules with a heatmap correlation coefficient ≥0.8 and p values < 0.01 and <0.001 were chosen and compared against each other to select unique and common genes between cell lines. Only unique cell line genes underwent the OTFBS analysis and only TF Families with the Z score (promoter) >3 were taken into further calculation. Here, the Z-scores are calculated with a continuity correction using the formula z = (x-E-0.5)/S, where x is the number of found matches in the input data, E is the expected value and S is the standard deviation (Genomatix). In each cell line, TF families from different cell groups (wt, HIF1kd, HIF2kd, HIF1 + 2kd) were compared to obtain TF families common in all analyzed cell groups and those which were found in at least one group. Common TF families were excluded from the analysis and only the top 10 (according to Z score (promoter)) of each cell group were used to conduct transcription factor-genes network. The TF families that were identified with the Genomatix TF enrichment analysis (TFEA) were broken down to those single transcription factors that made a TF family being called enriched by the TFEA tool. These enriched TFs and their regulated genes (also defined by Genomatix) were then used to conduct a regulatory network. Visualization and analysis of the network was done with Cytoscape 3.3.0^63^.

### GlobalNet and Pathway analysis

To get a more detailed view on the regulatory mechanisms underlying the investigated conditions, we further analyzed the TF-gene networks by emcompassing its intersection with GlobalNet – an unfocussed human protein-protein-interaction (PPI) network^22^. The resulting networks underwent a pathway analysis using the Genomatix GePS tool. For visualization of the interplay of the significantly enriched pathways in CKD development progression, we downloaded their pathway representations with the Bioconductor graphite package^64^. This package allowed us to access the BioCarta and NCI pathways as shown in Supplementary Table 7. All individual pathways were combined into a single network and imported into Cytoscape 3.3.0^63^. Node color represents the log2 expression foldchange, node size encodes the absolute value of the log2 foldchange, edge color represents the pathway. Directionality is based on pathway information and represented by arrows.

## Electronic supplementary material

Supplementary Data

Supplementary Table_1_2_3_4_5_6_7_8_9
