# Supplementary material for: Transcriptome-based network analysis reveals renal cell type-specific dysregulation of hypoxia-associated transcripts
Source: Sci Rep. 2017 Aug 17;7:8576. doi: 10.1038/s41598-017-08492-y (PMC5561250; doi:10.1038/s41598-017-08492-y)

Transcriptome-based network analysis reveals renal cell type-specific dysregulation of hypoxia-associated transcripts

Natallia Shved[1$], Gregor Warsow[2$], Felix Eichinger[3,] David Hoogewijs[4], Simone Brandt[5], Peter Wild[5,] Matthias Kretzler[3], Clemens D. Cohen[1, 6], Maja T. Lindenmeyer[1,6]

$ contributed equally

[1]Institute of Physiology and Division of Nephrology, University of Zurich, Zurich, Switzerland

[2]Department of Anatomy and Cell Biology, Universitätsmedizin Greifswald, Greifswald, Germany; Division of Theoretical Bioinformatics (B080), German Cancer Research Center (DKFZ), Heidelberg, Germany

[3]Department of Medicine, University of Michigan, Ann Arbor, Michigan, USA

[4]Department of Medicine / Physiology, University of Fribourg, Fribourg, Switzerland; Institute of Physiology, University of Zurich, Zurich, Switzerland

[5]Institute of Pathology and Molecular Pathology, University Hospital Zurich, Zurich, Switzerland

[6]Nephrological Center, Medical Clinic and Policlinic IV, University of Munich, Munich, Germany

Supplementary Figure 1 Bootstrapping distribution of the significantly GFR-correlated Genes in glomerular and tubular samples. Bootstrap correlation analyses were performed by selection of 10.000 sets of 83 randomly chosen genes and subsequent determination of the number of genes that correlated significantly with eGFR (correlation criteria: IρI >0.3; FDR-corrected $p<0.05$). This procedure has been applied individually to glomerular (A) and tubulointerstitial (B) batch-corrected gene expression data, respectively. (C) and (D): Number of significantly correlated genes (colums) and p-values (dots) generated for bootstrap samples with different sample sizes. For each size, ten bootstrap sample analyses were applied to 10,000 randomly chosen subsets of the 83 HIF-target genes. As expected, smaller datasets exhibit fewer significantly correlating genes, however, for each sample size tested, the number of correlating genes generated for the bootstrap samples was quite similar. Shown are the mean values for each measure, and error bars indicate standard deviation ((C) Glom, (D) Tub). Additionally, gender-specific correlation analyses were performed separately for females (E)/(G) and males (F)/(H).

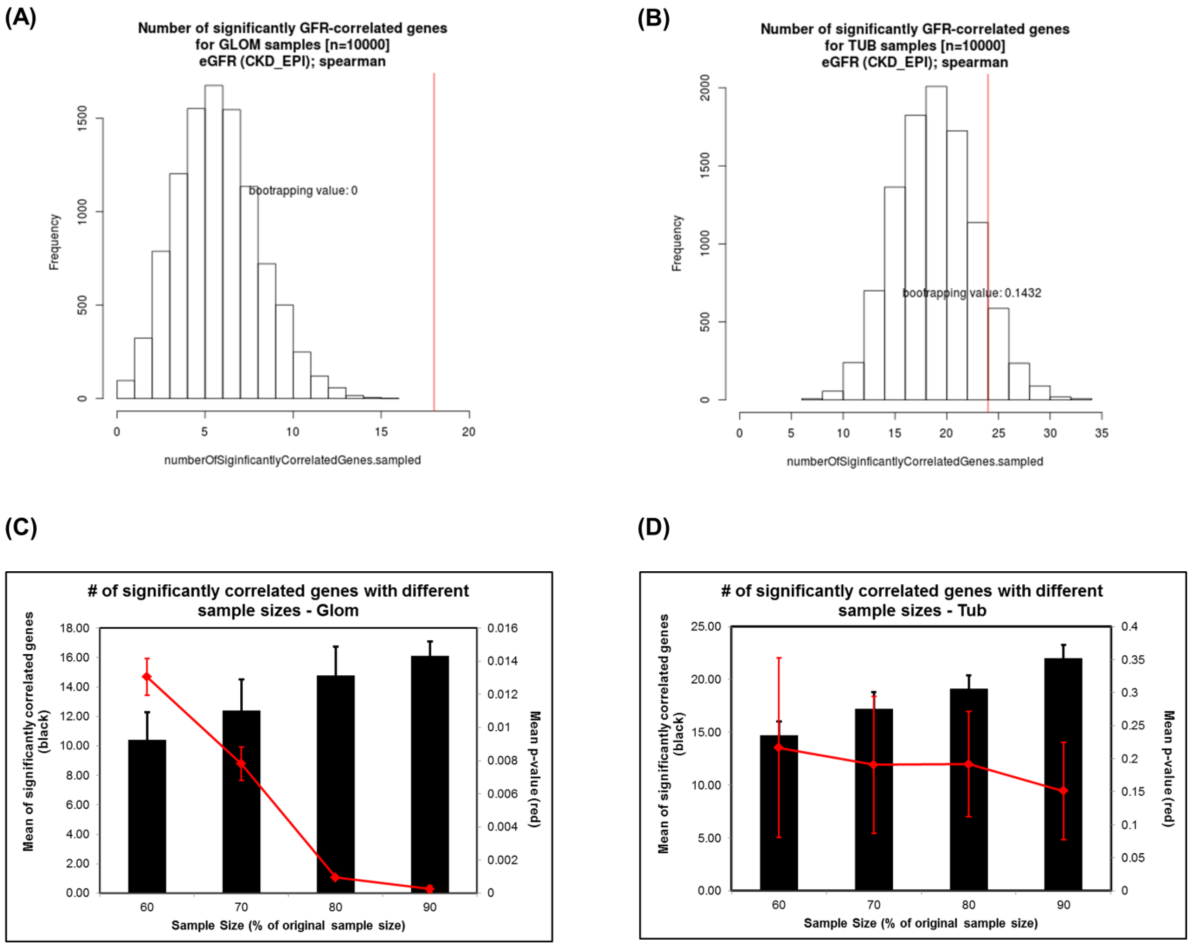

(E)

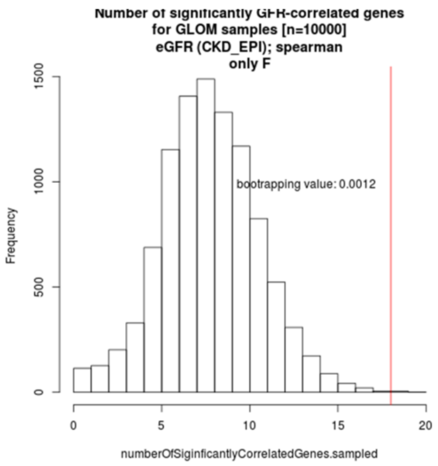
Number of significantly GFR-correlated genes
for GLOM samples [n=10000]
eGFR (CKD_EPI); spearman
only F
Frequency
bootrapping value: 0.0012
numberOfSiginficantlyCorrelatedGenes.sampled

(F)

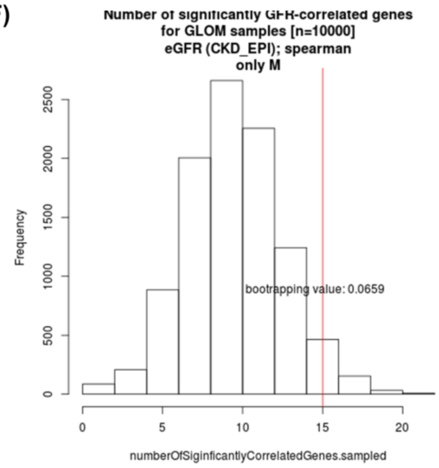
Number of significantly GFR-correlated genes
for GLOM samples [n=10000]
eGFR (CKD_EPI); spearman
only M
Frequency
bootrapping value: 0.0659
numberOfSiginficantlyCorrelatedGenes.sampled

(G)

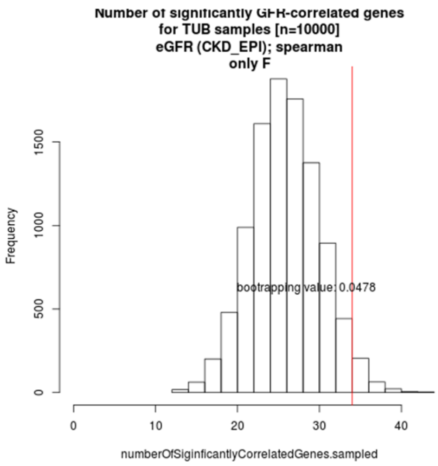
Number of significantly GFR-correlated genes
for TUB samples [n=10000]
eGFR (CKD_EPI); spearman
only F
Frequency
bootrapping value: 0.0478
numberOfSiginficantlyCorrelatedGenes.sampled

(H)

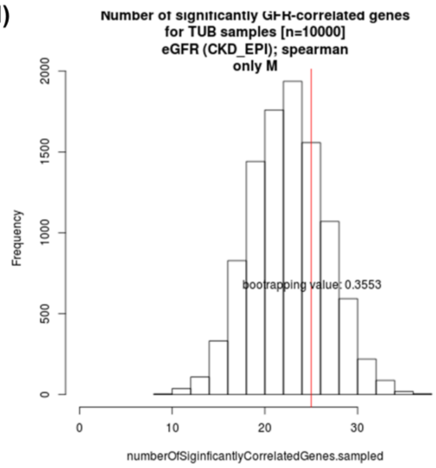
Number of significantly GFR-correlated genes
for TUB samples [n=10000]
eGFR (CKD_EPI); spearman
only M
Frequency
bootrapping value: 0.3553
numberOfSiginficantlyCorrelatedGenes.sampled

Supplementary Figure 2 Immunoreactivity for HIF1α, VEGFA, and ABCG2 in different stages of chronic kidney disease (CKD). (A) Association of eGFR (ml/min/1.73m2) and CKD stage in analyzed samples. (B) Histogram of the eGFR (ml/min/1.73m2). Cumulative bar charts comparing CKD stages with immunoreactivity for HIF1α in glomeruli (C), HIF1α tubulointerstitial (D), ABCG2 tubulointerstitial (E), and VEGFA tubulointerstitial (F).

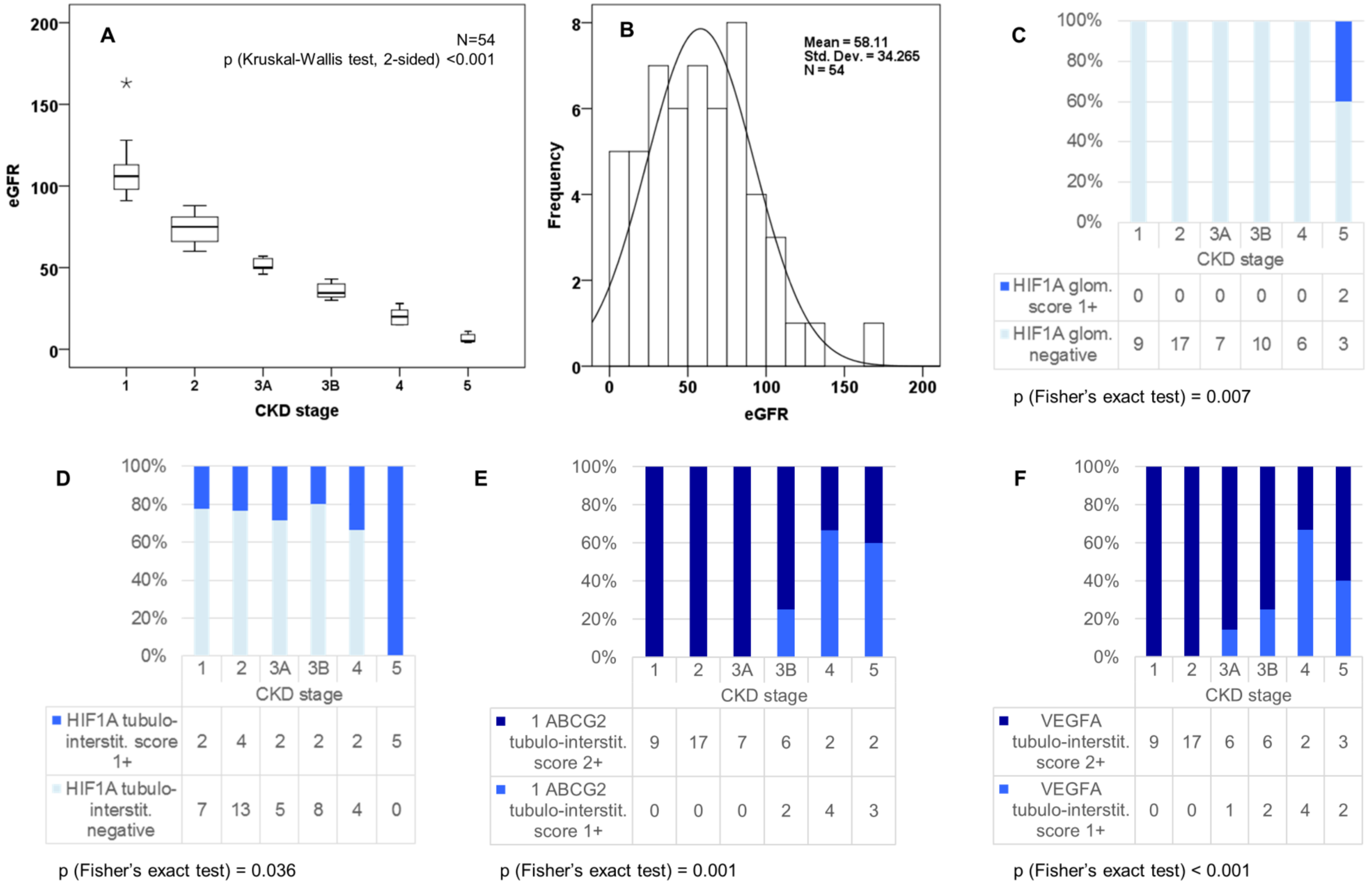

Supplementary Figure 3 Generation of stable HIF1α, HIF2α and HIF1α+HIF2α knock-down proximal tubular cells (HK2) and podocytes (AB81). Western Blot analysis of HIF1α and HIF2α in podocytes (AB81) and proximal tubular cells (HK2) (cropped blots, full length blots see page 13). Cells were subjected to 1% oxygen for 4h (HIF1α) and 24h (HIF2α) and whole-cell protein extracts were immunoblotted for the proteins indicated.

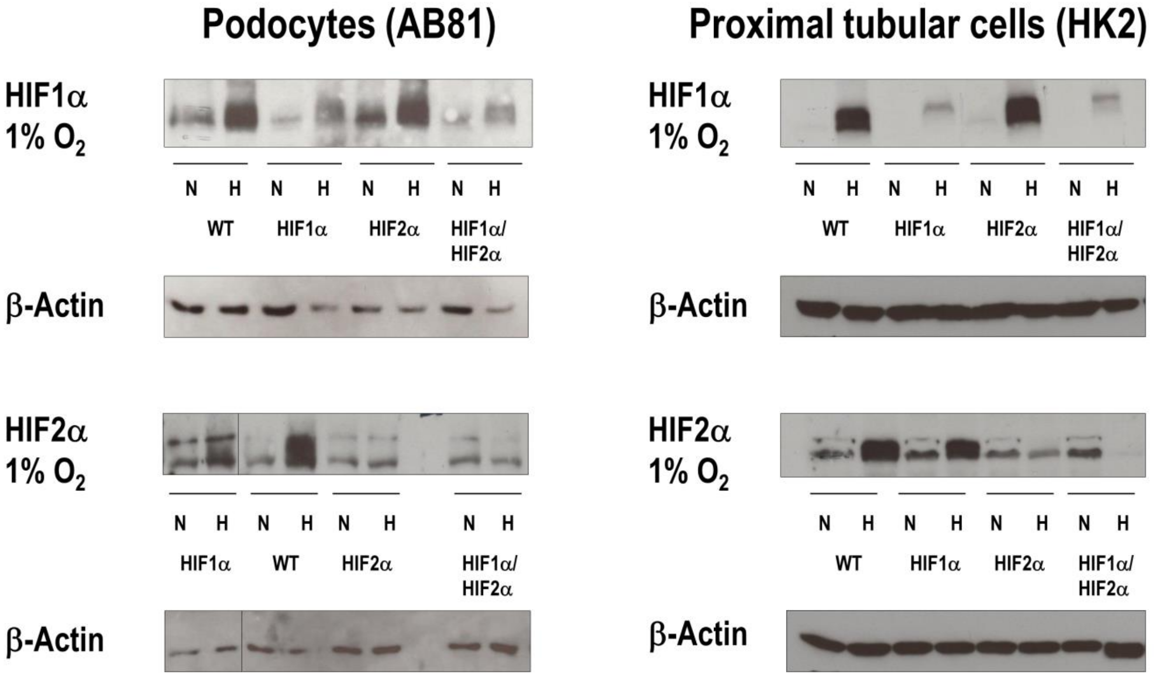

Supplementary Figure 4 Heatmap showing the correlation of the module eigengene (ME) to the respective knockdown condition, green means negative correlation, red means positive correlation. (A) Correlation heatmaps for the podocyte cell line (AB81) under hypoxia and normoxia. (B) Correlation heatmaps of the proximal tubular cell line (HK2) under hypoxia and normoxia. * p<0.05, ** p<0.01, *** p<0.001

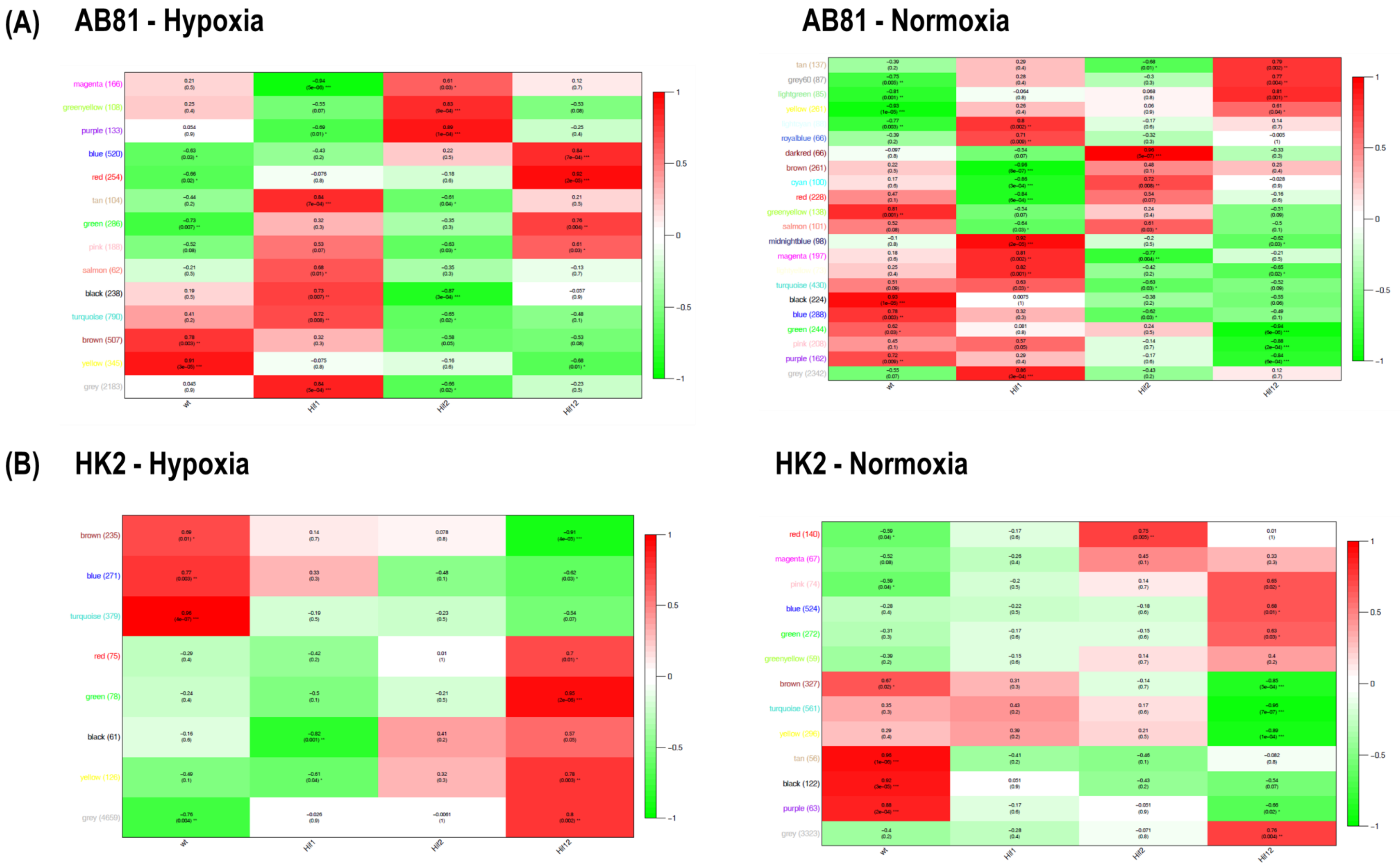

Supplementary Figure 5: GlobalNet Protein-Protein-Networks of the respective TFs and their regulated genes (under hypoxic/normoxic conditions) Comparison of the TF-gene-networks of each cell line with GlobalNet, a human protein-protein-interaction network resulting in protein-protein-interaction networks. Intersection of the different networks identified protein-protein-interaction networks that were exclusively related to podocytes (A) and (D), HK2 cells (B) and (E) or were enriched in both cell lines (C) and (F). Red nodes represent transcription factors, white nodes correspond to regulated genes.

(A) AB81 exclusive network - Hypoxia

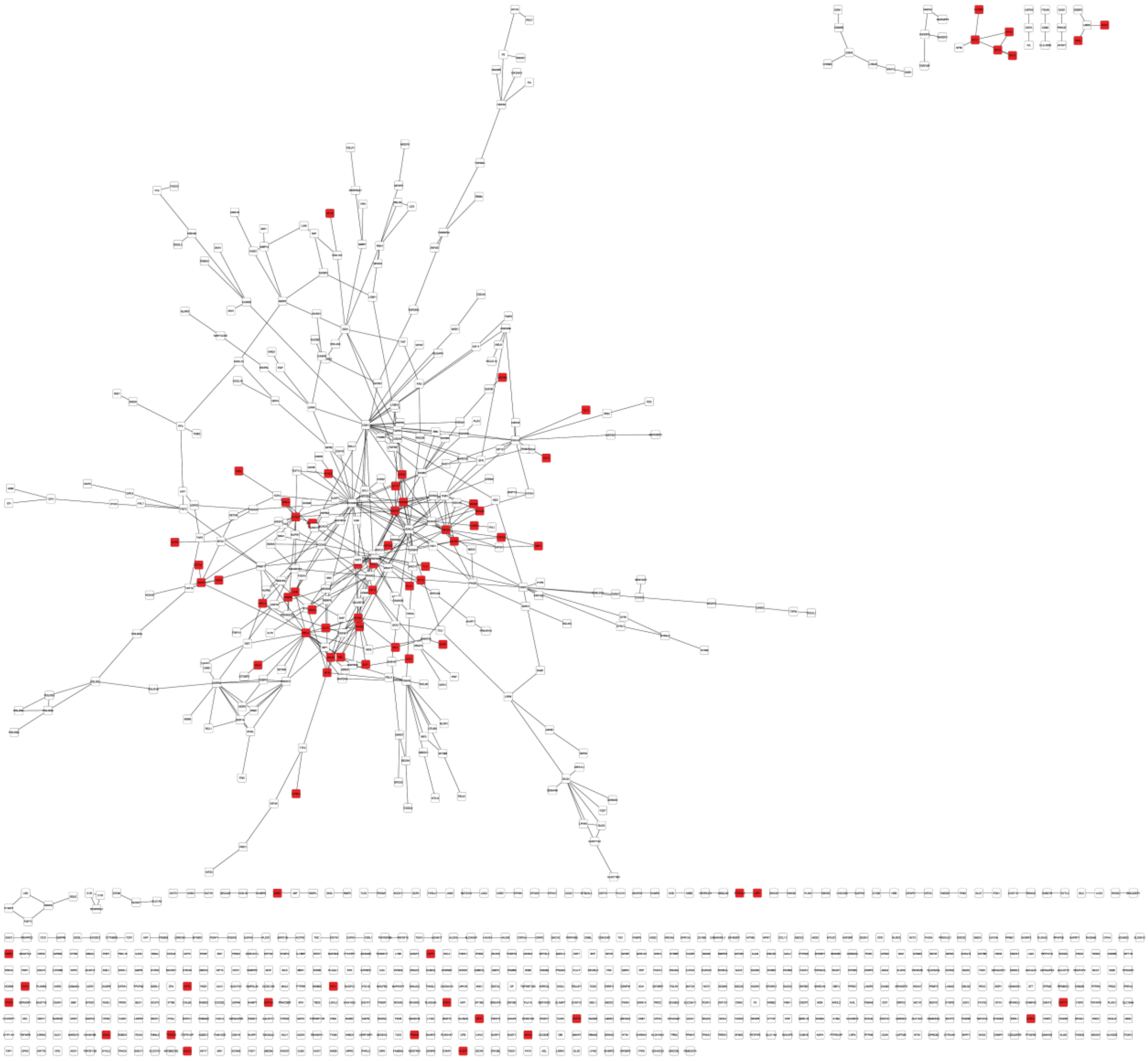

(B) HK-2 exclusive network - Hypoxia

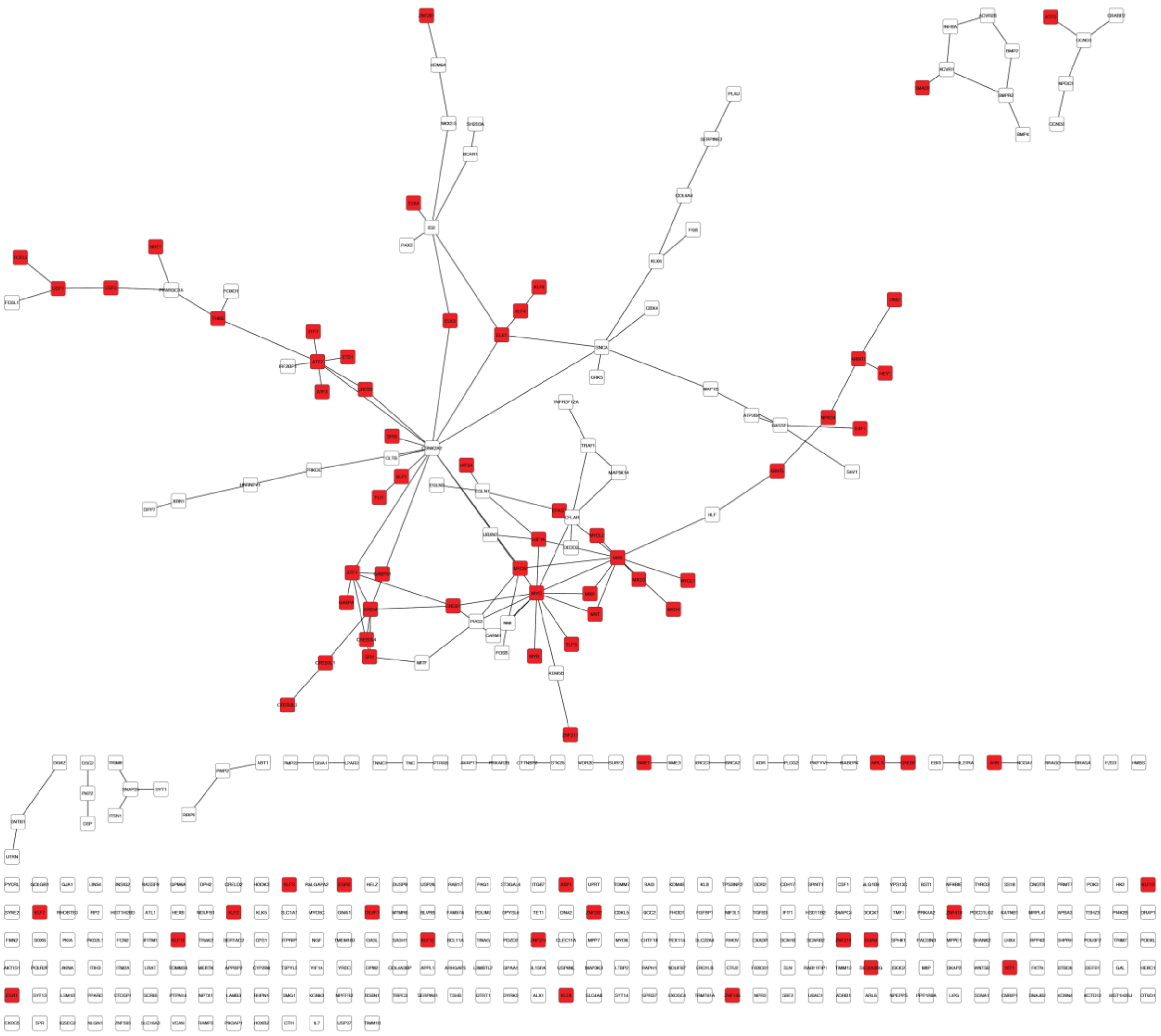

(C) AB81_HK-2 common network - Hypoxia

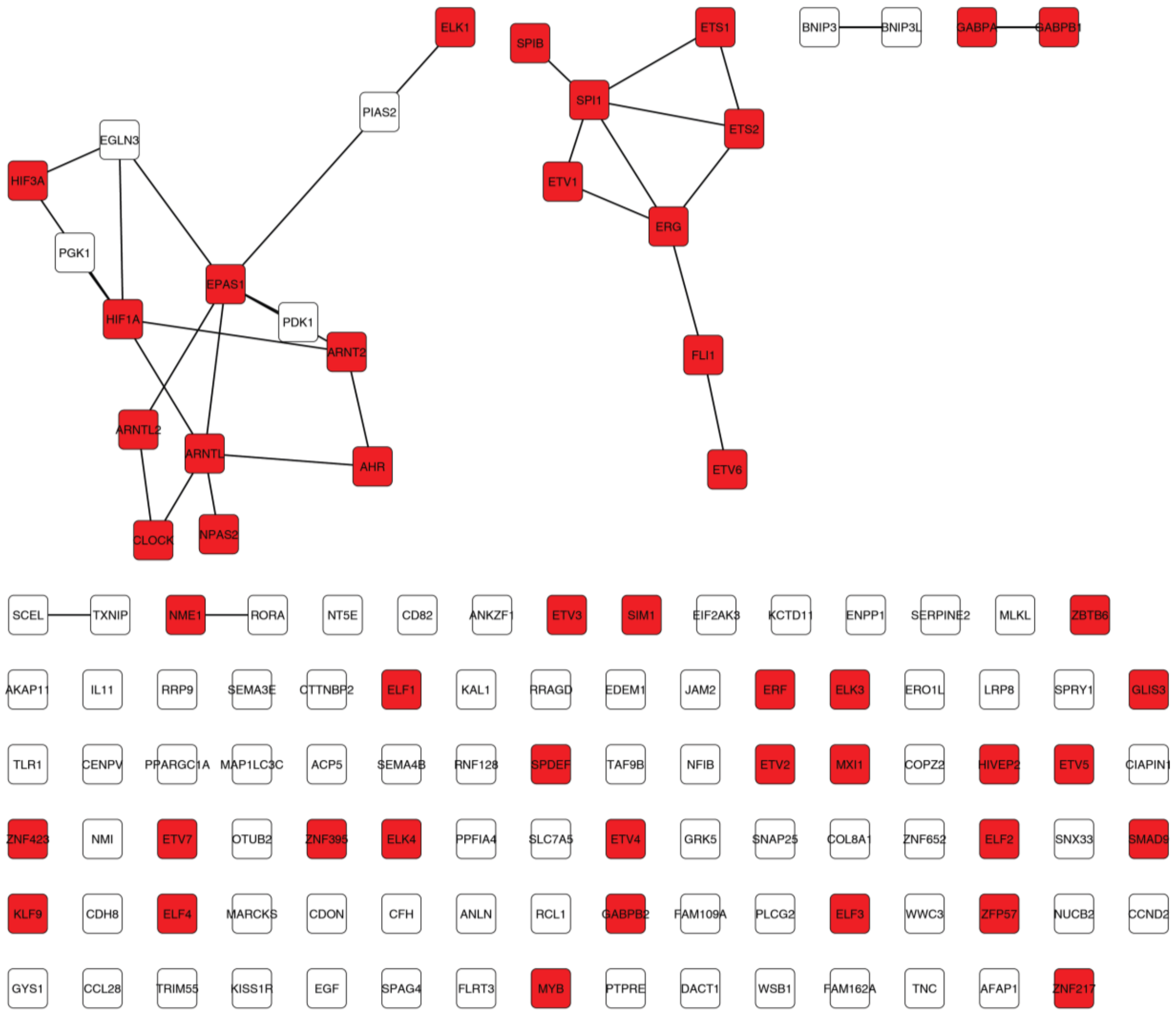

(D) AB81 exclusive network - Normoxia

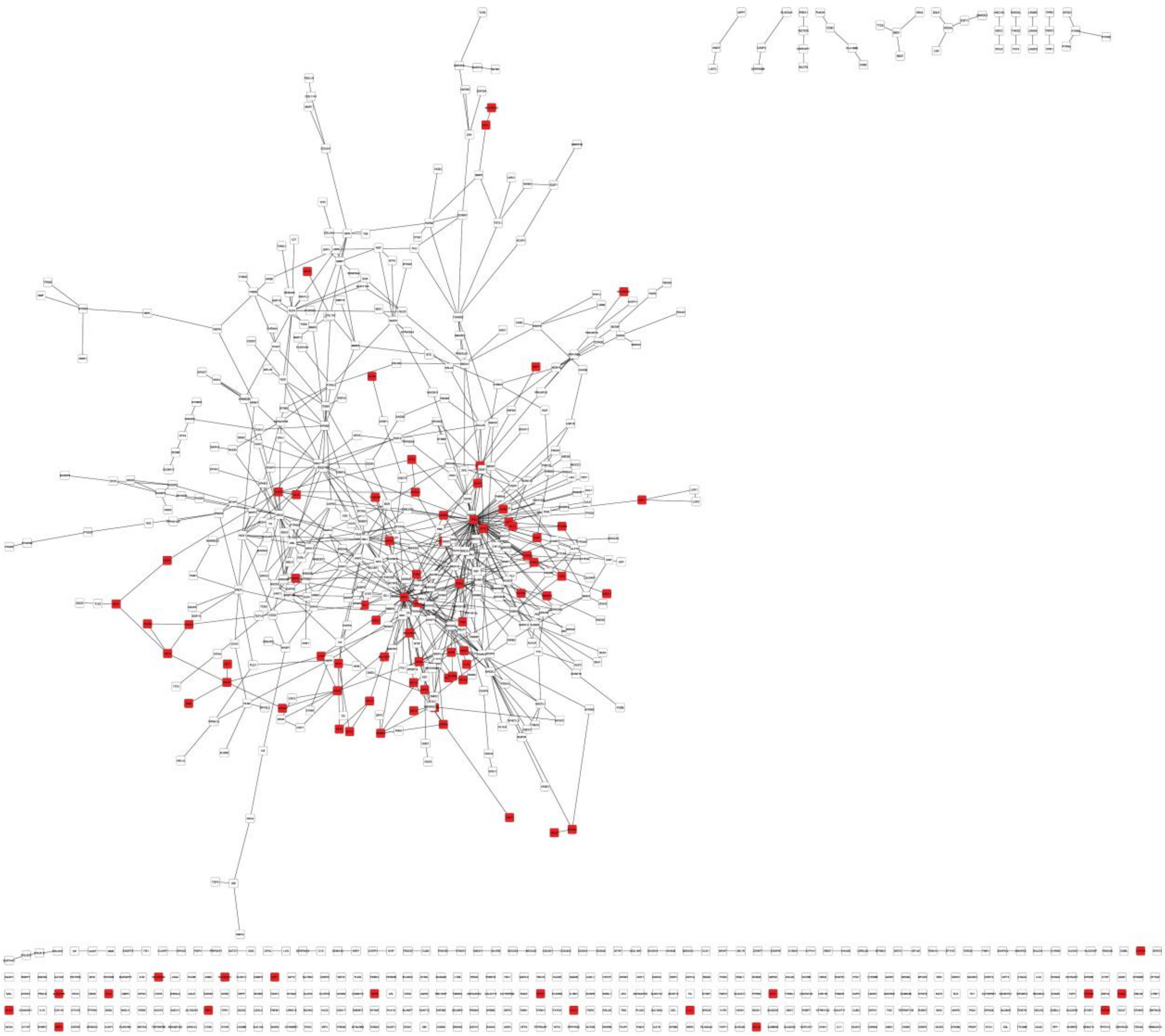

(E) HK-2 exclusive network – Normoxia

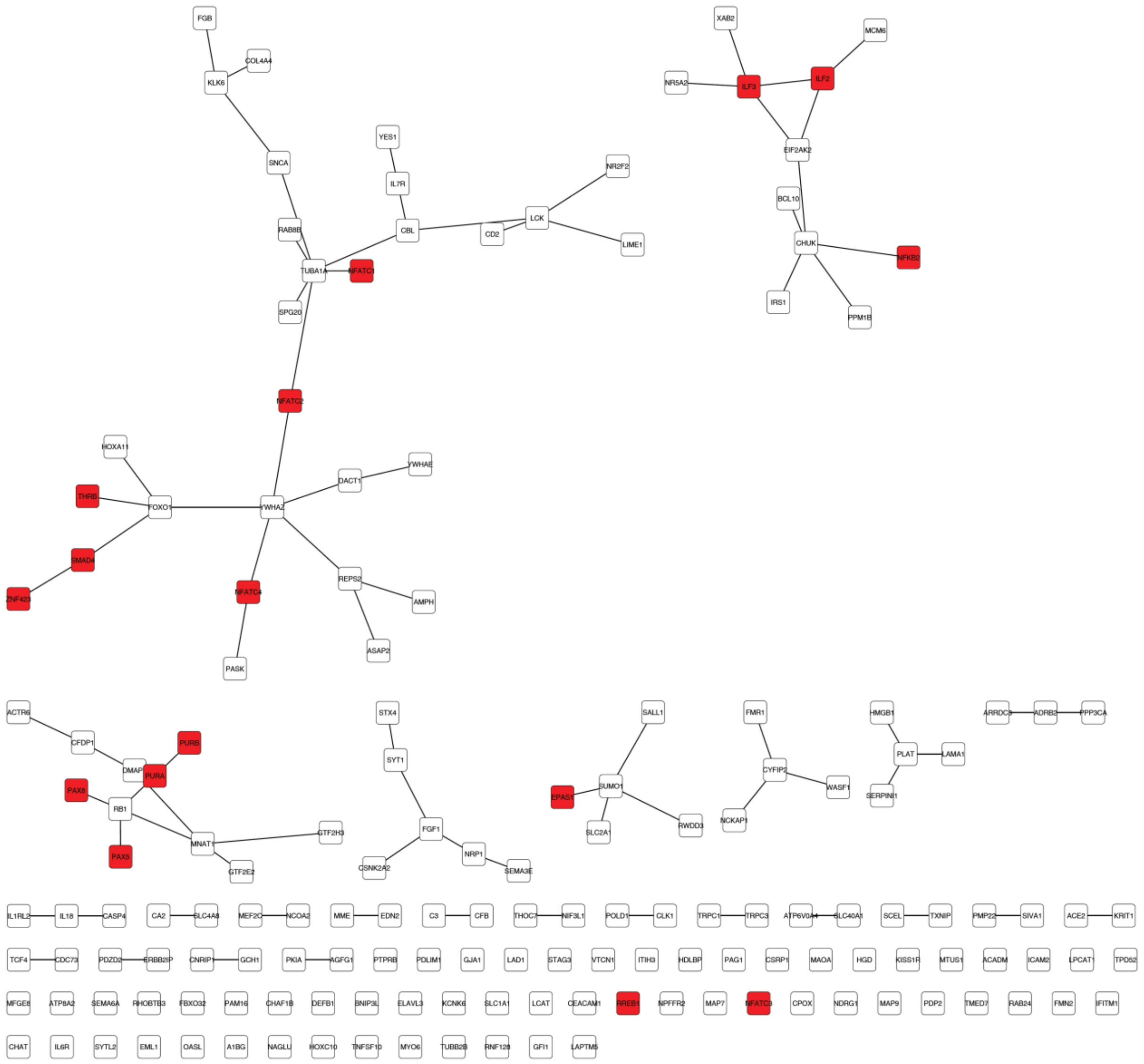

(F) AB81_HK-2 common network – Normoxia

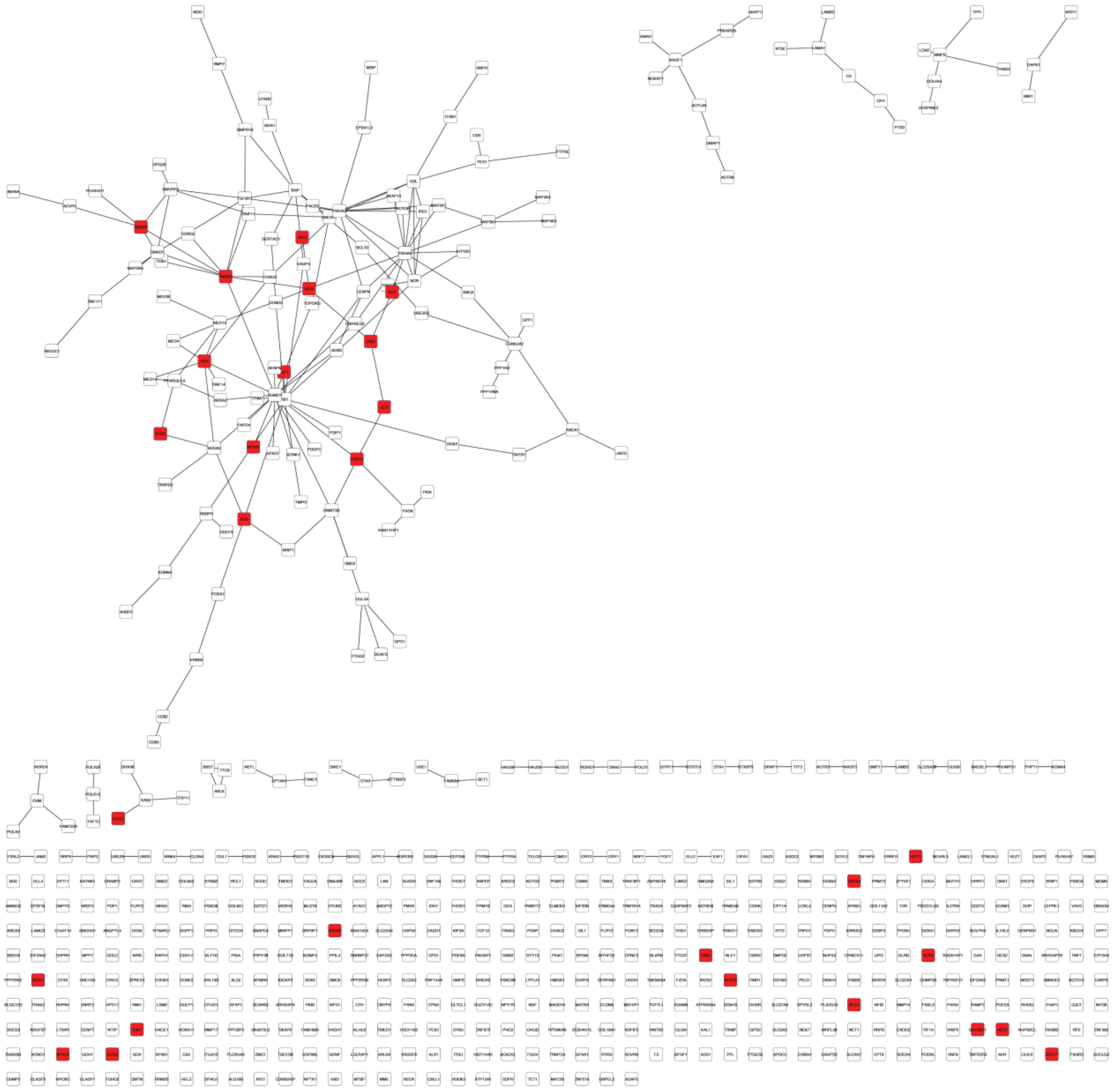

Full length Western Blot (Supplementary Figure 3) (A): HIF1α and β-Actin in podocytes. (B) HIF1α and β-Actin in HK-2 cells. (C) HIF2α and β-Actin in podocytes. The samples derive from the same experiment and gels/blots were processed in parallel. (D) HIF2α and β-Actin in HK-2 cells. Black rectangles indicate bands shown in the cropped blots in Supplementary Figure 3.

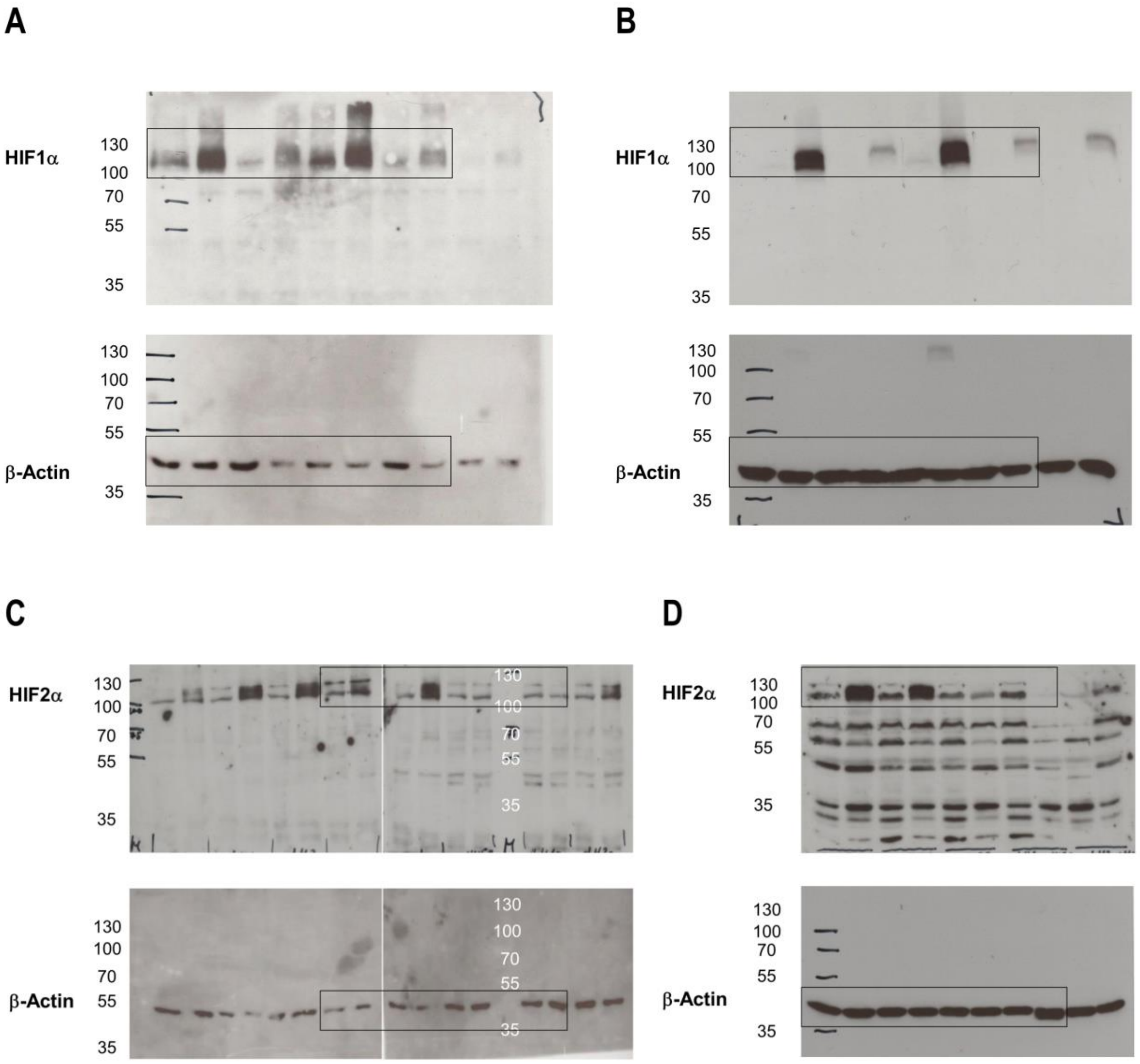

Supplement: Supplementary file 1 — Supplementary Data [file 41598_2017_8492_MOESM1_ESM.pdf]
